# Subtype-specific structural constraints in the evolution of influenza A virus hemagglutinin genes

**DOI:** 10.1038/srep38892

**Published:** 2016-12-14

**Authors:** Alexander P. Gultyaev, Monique I. Spronken, Mathilde Richard, Eefje J. A. Schrauwen, René C. L. Olsthoorn, Ron A. M. Fouchier

**Affiliations:** 1 Department of Viroscience, Erasmus Medical Centre, P.O. Box 2040, 3000 CA Rotterdam, The Netherlands; 2 Group Imaging and Bioinformatics, Leiden Institute of Advanced Computer Science, Leiden University, P.O. Box 9512, 2300 RA Leiden, The Netherlands; 3 Leiden Institute of Chemistry, Leiden University, P.O. Box 9502, 2300 RA Leiden, The Netherlands

## Abstract

The influenza A virus genome consists of eight RNA segments. RNA structures within these segments and complementary (cRNA) and protein-coding mRNAs may play a role in virus replication. Here, conserved putative secondary structures that impose significant evolutionary constraints on the gene segment encoding the surface glycoprotein hemagglutinin (HA) were investigated using available sequence data on tens of thousands of virus strains. Structural constraints were identified by analysis of covariations of nucleotides suggested to be paired by structure prediction algorithms. The significance of covariations was estimated by mutual information calculations and tracing multiple covariation events during virus evolution. Covariation patterns demonstrated that structured domains in HA RNAs were mostly subtype-specific, whereas some structures were conserved in several subtypes. The influence of RNA folding on virus replication was studied by plaque assays of mutant viruses with disrupted structures. The results suggest that over the whole length of the HA segment there are local structured domains which contribute to the virus fitness but individually are not essential for the virus. Existence of subtype-specific structured regions in the segments of the influenza A virus genome is apparently an important factor in virus evolution and reassortment of its genes.

Influenza A virus is an important pathogen, causing severe disease outbreaks in humans and domestic animals, in particular poultry and pigs^1^,^2^. The virus genome consists of eight RNA segments. Influenza A viruses are classified into subtypes, defined by antigenic properties of the hemagglutinin (HA) and neuraminidase (NA) proteins. Currently 16 HA subtypes (H1–H16) and 9 NA subtypes (N1–N9) are known to circulate in wild birds, which are the main reservoir of influenza A viruses^1^. Influenza A-like viruses of H17N10 and H18N11 subtypes, isolated from bats, have HAs and NAs substantially different from their homologs in classical avian influenza A viruses^3^.

Similar to other viral RNA genomes, the influenza A virus genome may encode RNA structures which play a role in virus replication^4^. The structures can be formed by the influenza virus genomic negative-sense viral RNAs (vRNAs) and complementary positive-sense RNAs (cRNAs) and mRNAs. Several functionally important structures have been predicted to fold in the central parts of influenza RNA segments, mostly in the positive-sense mRNA regions where a regulatory role of RNA folding is expected. In particular, functional RNA structures predicted near the splice sites of segments 7 and 8 and in the region containing start codons of alternate internal open reading frames of segment 2 were supported by phylogenetic analysis and shown to be folded *in vitro*^5^,^6^,^7^,^8^,^9^,^10^,^11^,^12^,^13^. Some of the conserved structures in segments 7 and 8 were shown to affect splicing^14^. Conserved structures were also suggested to fold in several domains of segment 5^15^,^16^.

A genome-wide analysis of influenza virus RNA segments suggested that negative-sense vRNA is globally less structured than the positive-sense counterpart^8^,^17^. This may be attributed to the functioning of vRNA in ribonucleoprotein (vRNP) complexes where most of the vRNA self-organized structure is melted upon nucleoprotein (NP) binding^18^,^19^,^20^. However, the absence of a global ordered RNA structure does not mean the absence of local structural motifs, and some predicted structured regions could be functional in both polarities^8^,^15^. Protein-free segment 8 vRNA has been shown to form stable conserved structures *in vitro*^21^. The functional importance of a pseudoknot structure folded in the packaging signal region of segment 5 vRNA has been demonstrated by compensatory mutagenesis using reverse genetics^15^.

Multiple functional structural elements are expected in the influenza vRNA. The efficient packaging of all eight vRNP segments requires a network of interactions, involving both the main packaging signals at the segment ends and the central domains^22^,^23^,^24^,^25^,^26^. The most likely mechanism of specific mutual recognition is pairwise interactions between RNA loops exposed from vRNPs^26^,^27^. The loops can be formed by local RNA structures that remain in vRNPs^18^,^28^ despite the regular superhelical global vRNP structure being dictated by multiple bound NP molecules^19^,^20^. Presumably, such RNA-RNA contacts were visible in electron tomography studies as string-like structures linking adjacent vRNPs within virions^22^,^24^. A loop-loop interaction between segments 2 and 8 has been shown to be required for efficient packaging of these segments into virions of the H5N2 subtype^25^. Remarkably, the networks of interactions required for efficient copackaging of genome segments seem to be considerably different in viruses of different subtypes^24^,^25^,^29^,^30^. Such differences may have a significant impact on virus evolution by reassortment of segments originating from different strains^26^,^30^,^31^,^32^.

In this work, we used a comparative RNA structure analysis to explore possible subtype-specific RNA structures in segment 4 (HA) of the influenza A virus genome, which encodes the important viral surface glycoprotein. Distinct subtypes of the HA protein exist (H1–H16), and enormous numbers of HA sequences from different strains, available from public nucleotide databases, can help in the detection of conserved structural motifs. In this analysis, we identified RNA motifs that impose significant structural constraints on the evolution of the HA segments of different subtypes. Such constraints are reflected in RNA-specific patterns of sequence diversity with coordinated variations (covariations) of the nucleotides paired in the conserved structures.

## Results

### Searching for potential conserved RNA structures within HA subtypes

From previous work in the search for structured regions in the influenza virus RNA segments^8^,^15^,^17^ it was anticipated that the conserved structural elements in the HA segment, if any, could be relatively small domains with moderate thermodynamic stability. The deviations from thermodynamically optimal structures, common in any large RNA molecule, are expected to be even more significant in the native influenza vRNA due to its functioning in the structure-destabilizing complex with multiple NP molecules^18^,^19^,^20^. Thus, the predictions obtained by algorithms for detection of conserved structures in related RNA sequences^33^,^34^, which are heavily based on RNA thermodynamics, are expected to be only partially correct in case of HA vRNA, which apparently lacks a global ordered structure^17^. On the other hand, functional significance of predicted local structural elements, irrespectively of their thermodynamic properties, can be estimated using analysis of nucleotide covariations that indicate significant evolutionary constraints and thus functional importance. Thanks to the availability of a large number of sequences from various influenza virus strains, mutual information calculations can help to identify significant covariations^15^.

Usage of different sequence datasets (see Materials and Methods) gave the opportunity to generate alternative secondary structure models for each of the HA subtypes. The differences between alternative models for a given subtype were variable, depending on intra-subtype similarity levels. For instance, in three predictions of H1 HA structures only 31% of predicted base pairs were common (149 out of 481), and 31–53% of predicted base pairs were the same in pairwise comparisons of the models, while in the predictions for less diverse H15 HA segments more than 99% of predicted base pairs were present in all three models. The overlaps between alternative models were considered as putative conserved functional local structures. In order to distinguish functional structural RNA motifs that exhibited significant constraints on HA sequences from potential artifacts of folding predictions due to sequence similarity of protein coding regions, the analysis of covariations in conserved local structures was performed.

The predictions of putative subtype-specific secondary structures by the RNAalifold and RNAz algorithms in HA segments, followed by mutual information calculations as described in Materials and Methods, yielded a relatively small number of significant covariations that could be attributed to RNA structural constraints. The significances were evaluated by ratios R_1_(xy) = M(xy)/H(x) and R_2_(xy) = M(xy)/H(y), which reflect correlations between substitutions of bases involved in putative base pairs, taking both mutual information M(xy) and variabilities (entropies) at two positions into account. Covariations with at least one correlation value R_1_(xy) or R_2_(xy) higher than 0.8 are listed in Table 1. These values correspond to correlations between substitutions of bases involved in putative base pairs, and such ratios of mutual information and positional entropies take the variabilities at two positions into account. Folding simulations on permuted HA sequence datasets (Supplementary Methods) showed that lower correlation values corresponded to p-values of 0.05 and higher. Covariations with a threshold of 0.5 for correlation values are given in Supplementary Table S1.

Three structures that contained more than one covariation characterized by a score of at least 0.8 were identified: in human H3N2 strains, in H7 viruses and in H15 viruses (Supplementary Fig. S1). The presence of multiple covariations may be considered as additional support for functional importance of the structures (see Supplementary Methods). Lowering the covariation threshold to 0.5 did not reveal any additional structures with multiple covariations in the RNAalifold and RNAz models.

### Multiple covariation transitions in the 794–839 domain of HA segments from human H3N2 viruses

**Figure 1:**
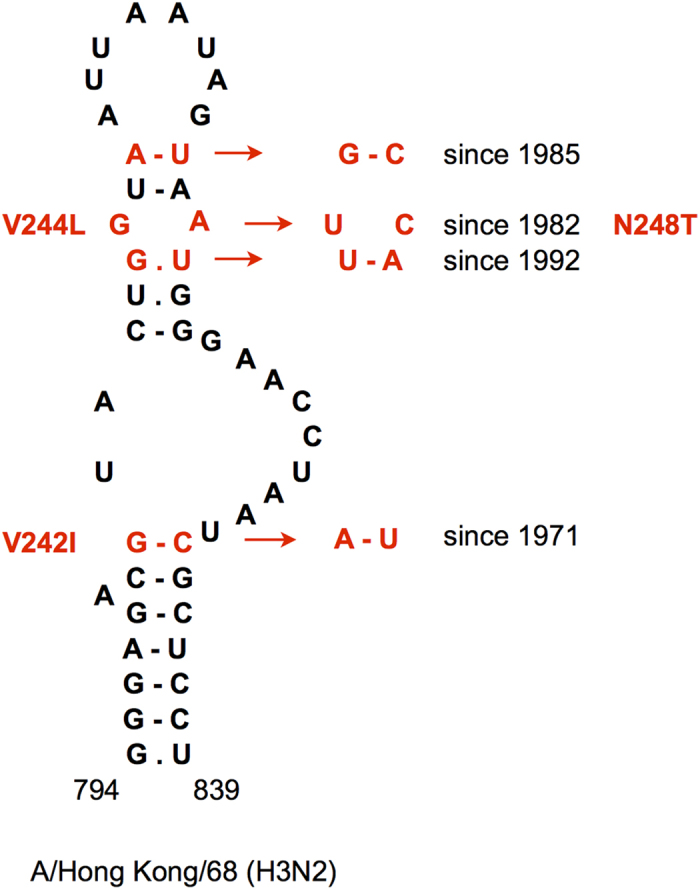
Predicted RNA structure in domain 794–839 of the HA segment of human H3N2 viruses. The sequence of influenza virus A/Hong Kong/68 (H3N2) is shown, representing the first H3N2 viruses in humans introduced in 1968. The main covariation events and amino acid changes caused by non-synonymous substitutions during subsequent evolution are shown in red.

Four covariations, each having a correlation value of at least 0.7, were identified in the stem-loop structure predicted in the region 794–839 of HA segments from human H3N2 strains (Supplementary Fig. S1, Fig. 1). The covariation transitions occurred in different periods of the evolution of these viruses (Supplementary Tables S2–S5). Two covariations (806–821 and 809–818) were determined by silent substitutions, while the substitutions of covariations 801–833 and 807–820 together lead to three amino acid changes (Fig. 1). The evolution of covarying nucleotides is punctuated, because a single change in a pair dominating over some period of time is quickly followed by a compensatory change defining a new dominating pair that is not prone to frequent changes (Supplementary Tables S2–S5). These covariation events did not exactly coincide with punctuated antigenic cluster transitions in the encoded HA protein^35^,^36^: the covariations and/or intermediate mismatch combinations with single substitutions first appeared in the clusters preceding those where the sequences with double changes dominate.

One of the covariations in the 794–839 domain (807–820) seems to be determined by non-canonical base pairing (Fig. 1). In the early human H3N2 strains (before 1975) the dominating combination is GA (Supplementary Table S4). The strains with the G807U change, resulting in a Watson-Crick UA pair, which circulated in 1974–1981, were in turn substituted by viruses with the secondary mutation A820C, resulting in the UC pair that dominates since its first appearance in 1982. This implies that GA and UC are the preferred combinations at this position, while the Watson-Crick UA pair is less favorable.

In a number of HA segments from human H3N2 strains, an alternative conformation of the 794–839 domain with the same bottom stem and different apical part has lower free energy (Supplementary Fig. S1). Furthermore, another conformation was predicted by the RNAalifold algorithm in H3 segments of avian strains, where the bottom helix exhibited a covariation at positions 801–833 between GC and AU, similar to human viruses (Supplementary Fig. S1). Although the correlation values computed for these positions in H3 segments of avian origin are less significant (R_1_(xy) = 0.56; R_2_(xy) = 0.48) than those in human strains (Table 1), this independent covariation event within the avian branch yields more confidence in the pairing of the 794–801/833–839 helix. Consistent with the different base pairs in the domain interior, no correlation between the changes at positions 806–821 and 807–820 were recorded in avian strains. In contrast, positions 809–818 in avian H3 segments did display an independent covariation (M(xy) = 0.26; R_1_(xy) = 0.42; R_2_(xy) = 0.44) between AU and GC, similar to human strains. Thus, in all H3 HA segments six independent covariation events are observed in this domain with its interior part apparently adopting alternative conformations (Supplementary Fig. S1).

### Various RNA structures in the HA cleavage site region are conserved in more than one HA subtype

**Figure 2:**
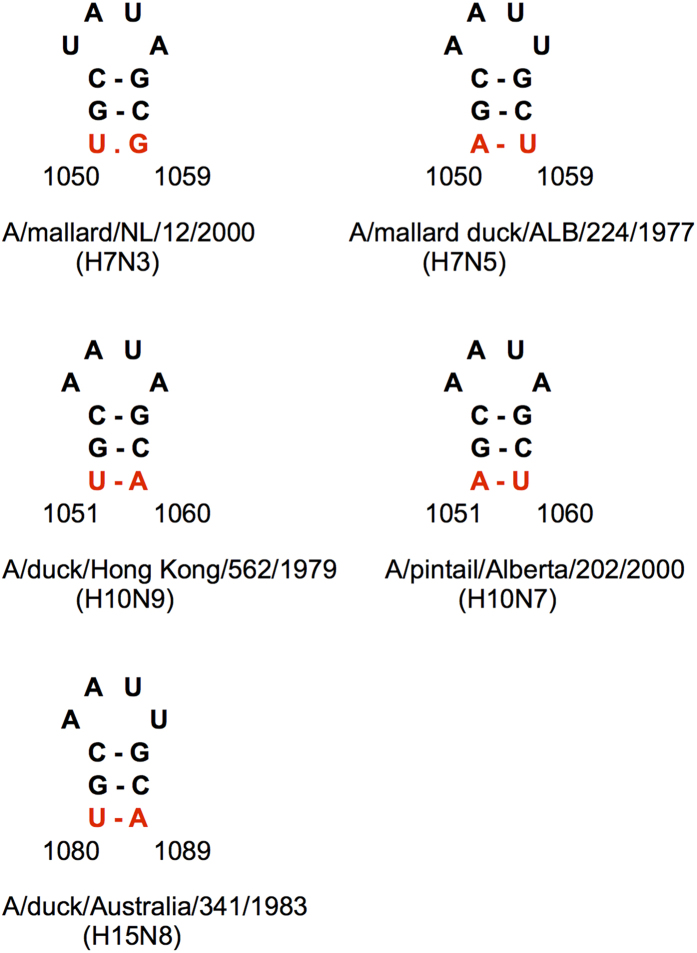
Independent covariation events (red) in the homologous hairpins predicted in the H7 and H10 HA segments. The hairpins in the representative strains of Eurasian and American branches of H7 and H10 subtypes are shown together with the homologous hairpin in the H15 HA segment.

**Figure 3:**
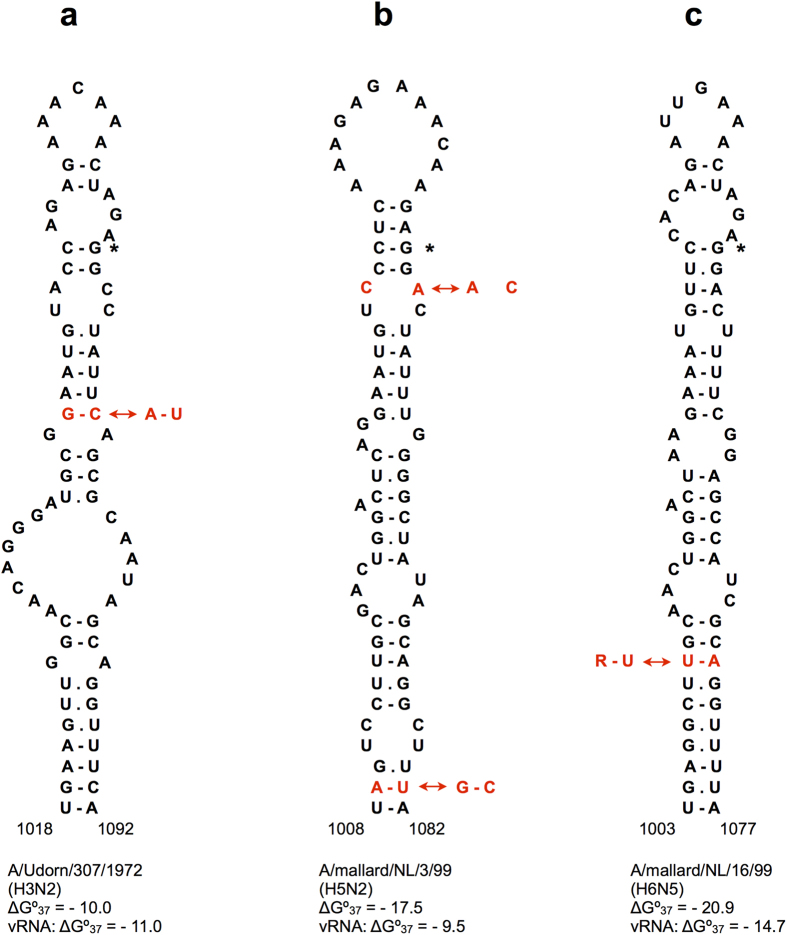
Homologous stem-loop structures in the cleavage site regions of H3 (**a**), H5 (**b**) and H6 (**c**) HA RNAs. The GGN codon coding for the first Gly residue of the HA2 chain downstream of the cleavage site is shown by an asterisk. Nucleotide covariations are shown in red. Folding free energies ΔG°_37_ (kcal/mol) are given for both positive sense RNA structures and corresponding negative-sense vRNA conformations. The covariation 1036–1057 in H5 HA RNA (**b**) involves two transversions between A.C and C.A mismatches in positive-sense mRNA, and these transversions correspond to the covariation between wobble base pairs G.U and U.G in vRNA. The 1010–1070 covariation in H6 HA RNA (**c**) also includes two transversions, R = A or G.

Two structures with homologs in different HA subtypes and covariations characterized by correlation values of at least 0.5 were detected using RNAalifold (Figs 2 and 3). Both are located in the central HA region, which encodes the amino acid motif required for HA polypeptide cleavage into HA1 and HA2 chains^37^.

One of these structures is a small hairpin in H7 and H10 subtypes, located 12 nt downstream of the nucleotides corresponding to the cleavage site. The H7 and H10 covarying positions are homologous to each other (Fig. 2) and undergo synonymous changes with the same continent-specific pattern: dominating UR in Eurasia and RU in America, where R is A or G (Supplementary Table S6). Not surprisingly, the hairpin is also possible in H15 segments, which form a monophyletic cluster together with H7 and H10^38^,^39^. Computed for the whole cluster, the correlation R_1_(xy) is 0.76, which is relatively high. Furthermore, it should be noted that the H7 and H10 covariations are separate events with covariation R_1_(xy) scores of 0.78 and 0.74 (Supplementary Table S1), respectively, lending more reliable support for the hairpin’s functional importance (Supplementary Methods).

Another structure, supported by covariations in different subtypes, was detected in H3 and H5 viruses, somewhat unexpectedly bearing in mind their distant phylogenetic relationship^38^,^39^. This stem-loop structure flanks the nucleotides encoding the HA protein cleavage site motif (Fig. 3a,b). The H3 structure, predicted in human H3N2 viruses, is supported by a covariation at position 1040–1073 (H3 numbering), characterized by one of the highest correlation values in this analysis: 0.90 (Table 1). The homologous H5 structure is detected in the negative-sense vRNA predictions by the RNAalifold algorithm but it is also thermodynamically stable in positive-sense RNA. It exhibits a weaker covariation with R_2_(x,y) = 0.59 at base pair 1036–1057 in H5 numbering (Supplementary Table S1), located closer to the hairpin loop as compared to the H3N2 covariation (Fig. 3a,b). All four positions, defining these two covariations in H3N2 human strains and H5 viruses, are wobbles in the HA codons, leading to silent substitutions.

High correlation values of the H3N2 1040–1073 covariation are determined by a fast transition from a GC pair to AU, occurring in 2002/2003 (Supplementary Table S7). Analysis of segment sequences and encoded HA proteins showed that the covariation occurred upon the transition between two antigenic clusters: SY97 and FU02^35^.

The H5 1036–1057 covariation is determined by two transversions converting YR typical for Eurasian strains into RY predominant in American H5 viruses (Supplementary Table S8), where Y is U or C and R is A or G. Due to frequent occurrence of both Watson-Crick combinations and mismatches AC or GU in this pair, its correlation values are not very high (Fig. 3b, Supplementary Table S8). On the other hand, within the American lineage a separate covariation event to the Eurasian-like UA pair was detected in 33 Californian strains of 2013 (Supplementary Table S8).

Free energy minimization structure predictions using the Mfold program^40^ showed that the hairpins predicted by RNAalifold in this domain (1036–1079 and 1036–1057 in H3N2 and H5 viruses, respectively) can be further extended into larger conserved stem-loop structures formed by homologous nucleotides in H3N2 and H5 viruses (Fig. 3a,b). This extended domain of 75 nucleotides is closed by an imperfect helix of 10 base pairs, interrupted by mismatches at different positions. The extension is significantly destabilized or unlikely to form at all in HA segments of many American H5 viruses (Supplementary Fig. S2). Furthermore, in some American strains alternative stem-loops, also flanking the cleavage site region, are more favourable (Supplementary Fig. S2). Nevertheless, the base pair between two wobble positions 1009–1081 in the conserved extended stem of H5 strains (Fig. 3b) exhibited a weak covariation (R_1_(xy) = 0.54), with an AU pair predominantly in Eurasia and GC in America (Supplementary Table S9).

Because of the conservation of this domain in such distant subtypes as H3 and H5, the potential for its folding was explored in all other HA subtypes. A similar extended stem-loop structure was found to be conserved in H6 strains, with an even more stable homologous bottom stem, mostly consisting of a perfect helix of 10 base pairs in positive-sense RNA (Fig. 3c). This domain-closing stem is also conserved within the monophyletic cluster consisting of H5, H6, H2 and H1 subtypes^38^,^39^. The domain interior base pairs are not conserved, leading to structural diversity with stem-loop structures in some H1 and H2 strains and a branched Y-shape in others (Supplementary Fig. S3). In many H1 and H2 viruses alternative structures have considerably lower folding free energies and are probably more favorable. In other subtypes, the folding of this domain is not conserved.

The folding of the domain bottom stem in the strains of the H5/H6/H2/H1 cluster (Fig. 3, Supplementary Fig. S3) is supported by a covariation in one of its base pairs (Fig. 3). It covaries within H6 subtype viruses (positions 1010–1070, R_1_(x,y) = 0.67) and exhibited even higher correlation values when computed for the whole cluster: R_1_(x,y) = 0.75 (Supplementary Table S10). Comparison of the dominant nucleotide combinations with the phylogeny of these subtypes^38^,^39^ suggests at least two independent covariation events at this base pair. One transition occurs within the H6 subtype, with the dominant UR substituted by RU in a branch of American strains, another separates H5 with its dominant UA from H1 and H2 that have predominantly RY (Supplementary Table S10). In the negative-sense H6 HA sequences, the bottom stem is less stable thermodynamically, and the RNAalifold predictions yield an alternative hairpin with a relatively strong covariation 1070–1094 (Supplementary Table S1).

### The conserved structure in the H5 cleavage site region is stabilized in the highly pathogenic A/goose/Guangdong/1/96 (H5N1)-like viruses

**Figure 4:**
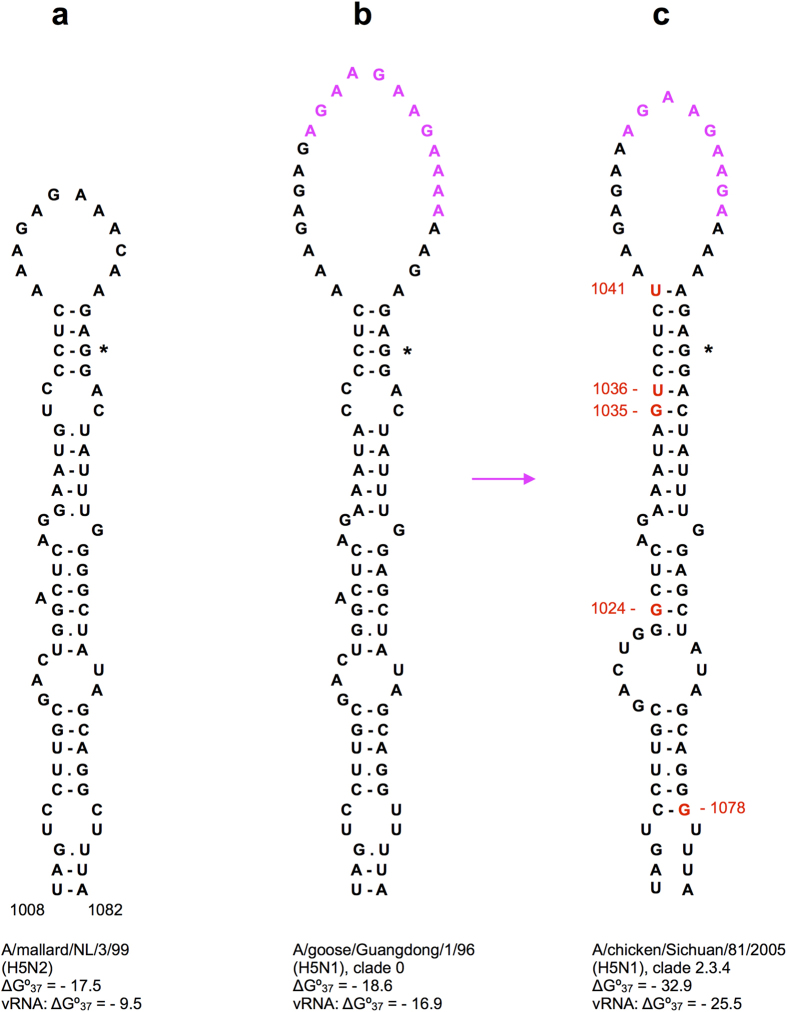
Stabilization of the stem-loop structure in the cleavage site region of H5 HA segments of the A/goose/Guangdong/1/96 (H5N1) lineage of highly pathogenic viruses. The GGN codon coding for the first Gly residue of the HA2 chain downstream of the cleavage site is shown by an asterisk. The codons inserted in the highly pathogenic H5 HA segments (**b,c**) are shown in magenta, structure-stabilizing mutations in red. The clades of the gsGD96 lineage are designated according to the accepted classification^41^. More detailed analysis of the stabilization of this structure in other clades is given in Fig. 5. Folding free energies ΔG°_37_ (kcal/mol) are given for both positive sense RNA structures and corresponding negative-sense vRNA conformations. It should be noted that the deletion of one of the four extra codons in some of the gs/GD/96 clades (compare (**c**) and (**b**)) stabilizes the structure by only 0.1 kcal/mol. The structure predicted in a low pathogenic H5 HA segment (**a**) is given for comparison.

Highly pathogenic avian influenza A (HPAI) viruses are characterized by multibasic cleavage motifs in the HA protein, which are created in H5 and H7 strains by inserting one or several codons coding for basic amino acids in the cleavage site region^37^. These insertions occur in the region corresponding to the hairpin loop of the predicted H5 subtype structure, so they are not expected to disrupt this folding (Figs 3 and 4). Remarkably, the structure is significantly stabilized further in the lineage of the most widespread H5 HPAI viruses that originated from the A/goose/Guangdong/96 (H5N1) strain (gs/GD/96) and that continue to circulate to the present day^41^.

The stem-loop structure in the ancestor gs/GD/96 HA segment, with an insertion of 12 nucleotides (Fig. 4b), has a folding free energy ΔG°_37_ of −18.6 kcal/mol in the positive-sense RNA, still comparable to the values calculated for homologous structures in low pathogenic avian influenza (LPAI) H5 HA genes of the lineages closely related to the gs/GD/96-like segments^42^. For instance, these structures in A/duck/Singapore/3/1997(H5N3), A/mallard/Netherlands/3/99(H5N2) and A/duck/Hokkaido/101/2004(H5N3) have free energies of −16.0, −17.5 and −22.4 kcal/mol, respectively. Despite its large loop of 24 nucleotides, the gs/GD/96 stem-loop structure is the lowest free energy local conformation for this HA sequence fragment, which is not observed in all H5 segments. The corresponding stem-loop in the gs/GD/96 negative-sense vRNA is also the thermodynamically optimal structure with a free energy of −16.9 kcal/mol.

**Figure 5:**
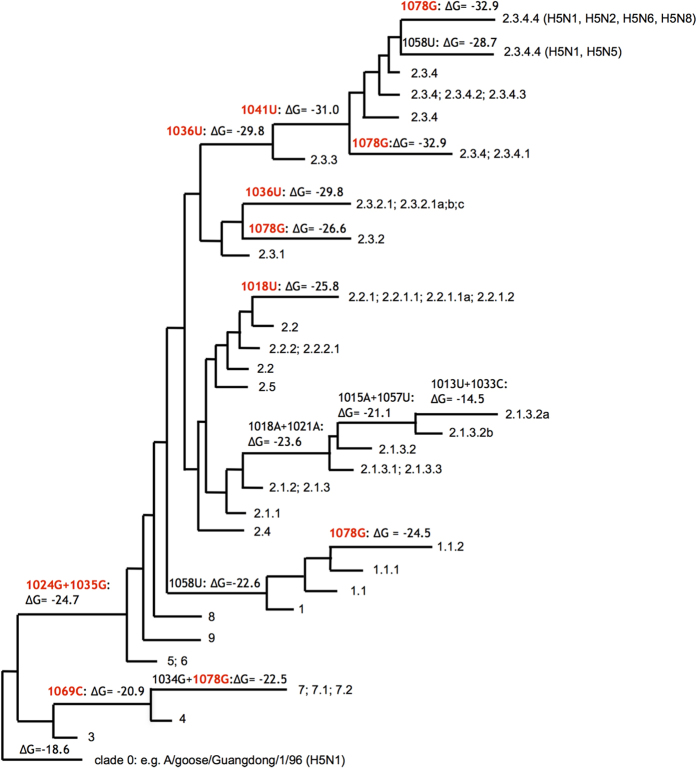
Stabilization of the structure predicted in the cleavage site region of H5 HA segments (Fig. 4) from the gs/GD/96 lineage of HP H5N1 strains. The gs/GD/96 phylogenetic tree^41^ is used in a simplified form to show the typical changes of the folding free energy ΔG°_37_ (kcal/mol) in the tree branches. Structure-stabilizing changes are shown in red. The clades are designated according to accepted nomenclature^41^, and the phylogenetic position of HP HA segments of reassortant H5N2, H5N5, H5N6, H5N8 strains in clade 2.3.4.4 is indicated.

Analysis of HA RNA sequences of the strains belonging to multiple clades that evolved from the gs/GD/96 lineage during 1996–2014^41^ showed that several stably inherited substitutions significantly stabilized the predicted stem-loop structure (Fig. 5). The most stable structures are formed in the segments of the currently most widely spread clade 2.3.4 strains that accumulated 5 substitutions leading to free energies of −32.9 kcal/mol (Fig. 4c). In particular, these 5 substitutions are observed in subclade 2.3.4.4 segments that are present in reassortant H5N8, H5N2 and H5N6 HPAI viruses isolated recently in Asia, Europe and America^41^. Structure-stabilizing substitutions were also observed in other clades of the gs/GD/96 lineage, the only exception being the clade 2.1.3.2 with several destabilizing mutations (Fig. 5). No extraordinary stabilization of RNA structures in the cleavage site region as compared to LPAI viruses was found in the H5 HA segments of the known HPAI outbreaks other than those caused by the gs/GD/96 lineage^43^.

### Multibasic cleavage sites of highly pathogenic H7 HA segments are also formed by insertions into hairpin loops

Similar to the insertions in the HA segments of HPAI H5 strains, the insertions in H7 HPAI segments seem to occur in the hairpin loops of LPAI H7 HA RNA structures. In the cleavage site region of H7 HA segments, different alternative structures were predicted (Supplementary Fig. S4). Close values of folding free energies of alternative stem-loop structures in this region suggest a possibility of conformational transitions, with different alternatives favored in various strains. The 1050–1059 hairpin, also conserved in H10 and H15 subtypes (Fig. 2), interferes with the extensions of these stem-loops, apparently stabilizing smaller domains (Supplementary Fig. S4). An equilibrium between alternative conformations is supported by involvement of nucleotide 1059 in two covariations: 1050–1059 within the 1050–1059 hairpin and 1020–1059 in the bottom stem of one of the stem-loop structures flanking the cleavage site region. The H7 HPAI HA stem-loop domains are mostly stabilized by the base pairs formed by inserted sequences (Supplementary Fig. S4) that may originate from recombination with other RNA molecules^43^.

### Weak constraints on the predicted structures in the minimal packaging regions

A few structures with significant covariations were revealed in the minimal packaging signals of the HA vRNA in virions. In H1 HA segments, these signals include both vRNA termini, containing 3′ and 5′ untranslated regions (UTRs) and extending to the coding part by 45 and 80 nucleotides from the 3′- and 5′-ends, respectively^44^. Among the vRNAs of all subtypes, only two hairpins with covariation correlation values of at least 0.8 were predicted in the regions homologous to these sequences: in the 3′ domain of H13 vRNA and in the 5′ domain of H14 vRNA (Supplementary Table S1, Supplementary Fig. S5). It should be noted, however, that the high correlation values of 1.0 obtained for covariations in all structures predicted in H14 HA segments (Supplementary Table S1) might be determined by the small number (N = 16) of available sequences. The structure with two covariations in the H15 3′ packaging signal region (Supplementary Fig. S1) was only predicted in positive-sense RNA sequences and was not present in the negative-sense vRNA predictions.

Lowering the correlation value threshold to 0.5 adds only one hairpin predicted in the 3′ packaging signal of H1 HA vRNA at positions 78–67 (Supplementary Table S1, Supplementary Fig. S5). Although its covariation at base pair 68/77 has a relatively low correlation value of 0.62 (statistically unreliable, with an estimated p-value of about 0.6), possible functional importance of the hairpin is supported by prediction of a homologous hairpin in the H2 vRNA, which, in turn, exhibits a weak covariation (R_2_(xy) = 0.31) at another base pair (Supplementary Fig. S5). Furthermore, a hairpin with a similar tetranucleotide loop sequence and a covariation with comparable correlation values was predicted in the H6 vRNA, but the position shifted 3 nucleotides downstream in relation to H1 and H2 hairpins according to the alignment of these sequences. The H13 vRNA hairpin is located 9 nucleotides downstream in the alignment as compared to that of H1 and H2 subtypes, but in distance from the vRNA 3′ end deviating by only one nucleotide from that of the H2 hairpin (Supplementary Fig. S5).

### Impact of conserved structures on virus replication

In order to study the influence of conserved RNA structures in HA segments on influenza A virus replication, mutant viruses were designed using reverse genetics. For these experiments, the following structures located in different HA regions were selected: domain 794–839 of H3N2 human strains (Fig. 1), the stem-loops flanking the cleavage site regions in H3N2 and highly pathogenic H5N1 viruses (Figs 3a and 4c), and the hairpin in the packaging signal region of H1 HA segments (Supplementary Fig. S5).

**Figure 6:**
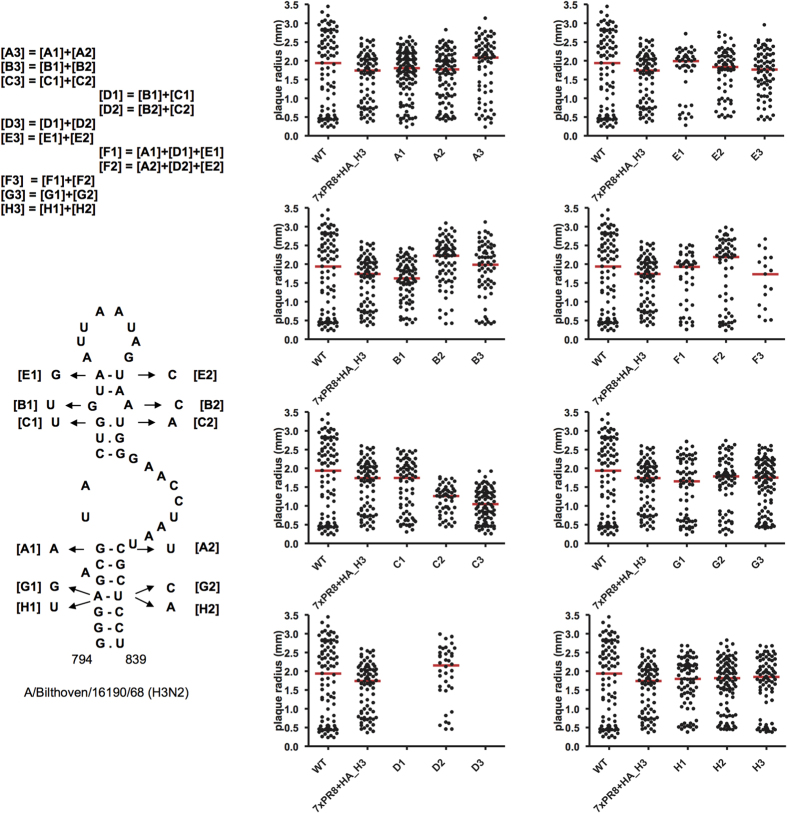
Mutagenesis of the predicted HA segment domain 794–839 of human H3N2 viruses. Plaque assays of recombinant viruses containing desired mutations were performed as described in Methods. The plots show the plaque radius for multiple individual plaques as a measure for virus replication. Dots represent individual plaques, red lines correspond to median plaque sizes. Point substitutions and their combinations in mutants are indicated in the domain structure. In each of the mutant series, structure-disrupting mutations with index 1 ([A1], [B1], etc.) were introduced to the left part of the structure, structure-disrupting mutations [A2], [B2], etc. were introduced to the right part, mutants containing the changes in both parts ([A3], [B3], etc.) were compensatory, restoring the structure. Mutant series A, B, C, E, G, and H affected one of the base pairs in the domain, mutant series D affected 2 pairs, and series F affected 4 pairs. Mutations [A1], [B1] and [B2] result in amino acid changes observed in natural strains, see Fig. 1, [C2] leads to N248K substitution (note that [C2] is silent in [D2] = [B2] + [C2], as observed in natural strains), other single nucleotide substitutions are silent. Despite three attempts to rescue the D1 and D3 mutants, no recombinant virus was produced. WT, A/PR/8/34 virus; 7xPR8 + HA_H3, recombinant virus with the A/Bilthoven/16190/68 (H3N2) HA segment.

The series of mutations, introduced into the studied structures, were designed to disrupt the predicted base pairs by substitutions that could also complement each other, restoring the predicted structures in double mutants (Fig. 6, Supplementary Figs S6–S8). All mutations introduced in the cleavage site region and packaging signal structures were silent for the encoded HA amino acid sequence, while some non-silent mutants of domain 794–839 reproduced natural mutations in H3N2 viruses. The replication of mutants was studied by plaque assays, which were previously shown to be sufficiently sensitive to reveal effects of structure disruption and reconstruction in the NP segment RNA^15^.

In the experiments with HA mutants, significant effects on virus replication were detected only for some of the substitutions in the 794–839 domain of H3 HA RNA (Fig. 6). In contrast to all other designed HA mutants, it was not possible to rescue mutants D1 and D3, and despite three attempts still no recombinant virus was produced. Thus the combination of mutations G806U and G807U (mutant D1) severely affected the virus, whereas each of these substitutions separately was not detrimental for virus replication (mutants B1 and C1, Fig. 6). The substitution G806U is silent, therefore it can be concluded that the synergistic effect of the two mutations is determined by RNA misfolding rather than by the amino acid change (V244L) due to the G807U substitution. The compensatory substitutions, introduced in the mutant D3, were not sufficient to restore virus viability. However, the F1 mutant which contained, in addition to D1 mutations, the substitutions G801A and A809G, replicated similar to the wild type virus (Fig. 6). Remarkably, these differences are consistent with the history of substitutions recorded in natural human H3N2 strains (Fig. 1): the combination of G806U and G807U was not observed before G801A and A809G had occurred.

No obvious correlation was found between the observed behavior of mutants and the introduced changes in codon usage according to available data on codon frequencies in human or influenza virus genomes^45^. In mutants replicating similar to the wild type virus, both the changes that substituted more common codons by more rare ones and *vice versa* were used. As far as D1 mutant is concerned, one of its substitutions (B1) replaced the relatively rarely used (in human genes) GUA codon (Val) by another rare one UUA (Leu), while silent substitution C1 replaced commonly used CUG Leu codon by less frequent CUU. This was also reflected in the estimate of heterologous gene expression in 293 T cells according to the Codon Adaptation Index (CAI), computed using the CAI calculator^46^ for the wild type and mutant domains 794–839: the wild type CAI value was 0.65, while it was slightly decreased to 0.63, 0.60 and 0.59 in B1, C1 and D1, respectively. As seen, the CAI decrease in D1 is mostly determined by the C1 mutation, so this value alone cannot explain the phenotype difference between D1 and C1. Predicted attenuating effect of changing two neighbor codons^47^,^48^ suggested the opposite trend in D1: according to the data on ratios between observed and expected codon pair frequencies in human genes^47^, the pair of mutated codons (CUU and UUA) is over-represented with a ratio of 1.402, while the wild type pair (CUG and GUA) is under-represented (0.854). Thus, although we cannot exclude some effects of codon substitutions, they are unlikely to be the reason of severe effects in D1 and D3 mutants, and it should be noted that influenza A viruses, attenuated by non-optimal codon compositions, are still able to replicate despite containing hundreds of de-optimized codons^48^,^49^.

Other designed HA mutants in this and other HA domains exhibited no significant replication deficiency which could be attributed to RNA structure disruption (Fig. 6, Supplementary Figs S6–S8). Even disruption of multiple base pairs in the structures did not result in noticeable effects. Significantly smaller plaque sizes, caused by substitution U821A in C2 and C3 mutants (p < 0.01 and p < 0.001, respectively) of the 794–839 domain in H3 HA segments (Fig. 6) were apparently caused by the resulting amino acid change N248K in the encoded HA protein, which was never observed in natural strains. The mutant D2 with the U821A substitution being silent in combination with A820C coding for the observed change N248T (Fig. 1), did not deviate from the wild-type (Fig. 6).

## Discussion

The nucleotide covariation analysis of conserved RNA structures in the HA segment of the influenza A virus genome, presented here, suggests a number of local structured domains exist, which are specific for certain HA subtypes only and are not conserved in all 16 known subtypes. In contrast to the NS, M and NP segments, where several structures conserved in all influenza A strains have been identified in the coding regions^5^,^8^,^15^, no universally conserved structure is found in HA segments except for the well-known panhandle structure at the termini^4^.

A given covariation can serve as reliable evidence for a structural model only if it is likely that the observed bias at the putative base pair is indeed determined by RNA structure rather than by other evolutionary pressures or chance alone. Evaluation of covariation-based evidence for functional coevolution of two sites in a biopolymer is not straightforward^50^,^51^,^52^. A number of statistics have been proposed that measure how different the observed pattern of nucleotide pairs is from the expected upon independent changes at each of the positions. It is not the goal of this study to evaluate these measures, because they all have similar flaws in distinguishing functional coevolution from independent changes occurring in particular clades^52^. The metrics M(xy)/H(x) and M(xy)/H(y), which normalize mutual information values with the entropies of two sites, were chosen to evaluate the extent of structural constraint in putative covariations in assumption that the sites were paired indeed. The behavior of these metrics in datasets of well-established tRNA and rRNA structures was studied in detail^53^, allowing us to make comparisons. Folding simulations using datasets of permuted H2 HA sequences showed that single covariations with correlation values of less than 0.8 corresponded to p-values higher than 0.05 in structure predictions and thus did not provide sufficient support for base pairing (Supplementary Methods). As many base pairs in functional structures are not expected to have better correlations^53^, the conserved structures cannot be reliably identified by correlation values of single covariations.

An inadequacy of a covariation metric alone in the discrimination between different mechanisms of nucleotide coevolution can be compensated by taking the phylogenetic history of covariations into account^50^,^51^,^52^,^53^,^54^. Correlated changes in a putative pair occurring independently in more than one branch of a phylogenetic tree provide significantly more confidence in the base-pairing constraint. The same is true in case of covariations at different base pair positions in a given RNA structure element. The estimates based on permuted HA sequences show that even two independent covariation events with inconclusive individual covariation scores may serve as a strong support for a structural model (Supplementary Methods).

Indeed, several RNA structures supported by more than one covariation event were predicted in HA segments, pointing to important constraints determined by secondary structure. Interestingly, the most reliably supported structures were detected in the central regions of the HA segment, in particular, the region coding for the cleavage site motif in the HA protein. These structures turned out to be conserved in different HA subtypes, with independent covariations occurring at homologous positions.

On the basis of covariation data it is impossible to conclude whether predicted local hairpins are functional in the positive-sense RNA (mRNA or cRNA) or negative-sense vRNA, and folding free energy differences cannot be used in support of a conclusion because of the influence of NP binding on RNA structures within RNPs^18^,^19^,^20^. The HA vRNA hairpin loops in all regions of the segment may be involved in loop-loop contacts with other segments required for efficient copackaging in the virions^25^,^26^,^27^. Strong conservation of these hairpins in some HA subtypes and their disappearance in others is consistent with frequently observed non-random reassortment of compatible segments^26^,^30^,^31^,^32^. In positive-sense mRNA, locally stable structures may be involved in modulation of protein folding, with their folding free energies slowing down the ribosome movement and thus facilitating correct cotranslational formation of native domain structures^55^, or in interference with host innate immunity^56^,^57^.

The insertions coding for multibasic cleavage site motifs in the HA segments of highly pathogenic H5 and H7 viruses occur in the hairpin loops of stem-loop structures flanking the cleavage site region so that the folding topology remains the same (Fig. 4, Supplementary Fig. S4). Furthermore, in the worldwide circulating gs/GD/96 lineage of highly pathogenic H5N1 avian influenza viruses, the destabilizing effect of the increased hairpin loop size is compensated by mutations stabilizing the double-helical part of the structure. Apparently, maintaining a stable folding of this domain is important for virus fitness. The structured character of this HA segment region is also predicted by statistics of folding free energies and codon conservation^8^. Various local structures in this and adjacent regions were previously suggested to regulate sequence duplications by transcriptional slippage on polypurine sequences in highly pathogenic H5 segments^58^,^59^.

The conserved H5 and H7 structures in the central HA domain (Fig. 4, Supplementary Fig. S4), folded either in positive-sense cRNA or negative-sense vRNA, may be an important factor affecting sequence insertion and recombination that lead to a pathogenic phenotype. The location of insertion sites in the hairpin loops is suggestive for a recombination mechanism with hairpin-mediated template switching, resembling the copy choice mechanism in retroviruses^60^. On the other hand, transcriptional slippage of a DNA-dependent RNA polymerase was shown to be stimulated by nascent RNA hairpins located near the slippage site^61^, and a similar topology was suggested to explain RNA editing by the RNA-dependent RNA polymerase of Ebola virus^62^. Stabilization of the H5 HA structure in the gs/GD/96 lineage years after the slippage has occurred and the covariation in the homologous fold in human H3N2 viruses (Fig. 3, Supplementary Table S7) suggest that this stem-loop structure has also some function which is not directly related to evolving multibasic cleavage site motifs.

A small hairpin, predicted to fold in the region essential for vRNA packaging (Supplementary Fig. S5), exhibited only weak constraints in covariations despite the conservation of its motif in different subtypes. Such a pattern of independent covariation events indicates a functional role of the hairpin which either has a redundant function or can tolerate mismatches in the stem. Plaque assay experiments with mutated hairpins did not reveal any effect of this structure on virus replication (Supplementary Fig. S8). This is similar to the results obtained on packaging of NS segments with randomized packaging signal sequences, showing that sequences without any obvious sequence or structure similarity to the wild-type were sufficient for segment incorporation^63^. However, this is in contrast to the complementary mutagenesis of the structures predicted in the NP segment packaging signal, which showed their importance for efficient virus replication^15^.

A strong effect of the specific combination of mutations in the 794–839 domain of human H3N2 viruses on virus viability (Fig. 6) seems to be determined by a misfolded RNA conformation prohibiting HA segment functioning rather than by just disruption of the conserved structure. Other structure-disrupting combinations of mutations did not impair virus replication. Plaque assays of mutant viruses with disrupted or destabilized structures predicted in the HA cleavage site region also did not reveal any replication defects (Supplementary Figs S6 and S7). Similar inconsistency between evolutionary conservation of virus structures and their influence on virus replication assays was previously noted for HIV-1 and murine norovirus^56^,^57^. It has been suggested that RNA folding could have a function that contributes to virus fitness and persistence in nature but is not essential for basic replication under the experimental conditions. For instance, disruption of murine norovirus RNA structures was shown to attenuate the virus *in vivo* while no effect in cell culture was seen^57^. One of the possible mechanisms is an interference of virus RNA structure with host innate defense responses^56^,^57^. It is also possible that the role of a single hairpin involved in the network of multiple contacts between genome segments^24^,^25^,^26^ is redundant.

Certain correlations between covariation events in the structures of HA segments from human H3N2 viruses (Figs 1 and 3) and HA cluster transitions in antigenic evolution in these viruses^35^,^36^ suggest that punctuated evolutionary trajectories of RNA and encoded protein may influence each other. Thus, in the 807/820 covariation from GA to UC the first G807U change is apparently favorable because of the cluster-difference V244L substitution in the HA protein^35^, while the compensatory A820C change seems to be caused by the need to restore the original RNA structure, leading to another amino acid substitution. The co-occurrence of the 1040/1073 covariation (Fig. 3a) with the SY97/FU02 cluster transition might be a coincidence, because it involves two silent substitutions occurring almost simultaneously on the evolutionary timescale, leading to a punctuated nature of RNA structure evolution resembling that of antigenic HA properties^35^,^36^.

Presumably, RNA secondary structures displaying nucleotide covariations only partially represent functional RNA folding in the influenza A virus HA segment. The covariation analysis reveals the importance of RNA folding in the HA segment evolution, but the most conserved RNA structures could be hidden from this consideration in genome regions that are less prone to mutations. Predictions of consensus RNA structures by the RNAalifold algorithm contained a number of potential hairpins with (nearly) invariant stems within a given HA subtype. Such hairpins could be formed by sequences containing clusters of conserved HA codons that are thought to determine the segment packaging signals^29^. In the absence of sufficient sequence variation, predictions of these hairpins are not reliable, and different alternative structures are possible. Diverse patterns of highly conserved codons in different HA subtypes^29^ suggest that such structures would be subtype-specific, similar to those supported by covariations. Multiple subtype-specific structural motifs in vRNAs could determine specificity of intersegmental RNA-RNA interactions upon vRNA packaging and therefore the selection of specific constellations of compatible segment variants during reassortment^26^,^30^,^31^,^32^. Elucidation of RNA folding patterns in the influenza A virus genome will significantly contribute to our understanding of the evolution and reassortment of the virus genes.

## Methods

### RNA structure predictions

Initial predictions of potential structured RNA domains in the influenza A virus HA segments were carried out using two algorithms: RNAalifold^33^ and RNAz^34^. The RNAalifold algorithm calculates the secondary structure optimal for a dataset of aligned RNA sequences on the basis of the predicted ensemble of low free energy conformations. RNAz predicts locally optimal structures using a scanning window of the RNA alignment, estimating deviations in these structures from the patterns expected by chance alone. Conserved structures in a given HA subtype were identified by comparisons of alternative predictions yielded by different datasets of representative strains using the method applied previously for the NP segments^15^. Details are provided in the Supplementary Information. Essentially, for every subtype three different datasets of representatives were used for RNAalifold and RNAz predictions. Local structures that were present in at least two out of three RNAz and/or RNAalifold models and exhibited putative covariations in the representative sequences were further considered for evaluations of covariation significance.

### Evaluation of covariation significance

Significance of covariations was first evaluated by mutual information calculations using all available sequences of the HA segment of a given subtype, taking positional entropies of sequence alignment into account, as described previously in the study of NP segment structures^15^. Details are provided in the Supplementary Methods. For a putative covariation of nucleotides at positions x and y, the ratios of mutual information M(xy) to positional entropies at the two positions R_1_ = M(xy)/H(x) and R_2_ = M(xy)/H(y) were calculated. In the estimates of covariations in RNA structures, these measures are less sensitive to dataset bias than M(xy) scores^15^,^53^. The confidence levels for single and multiple covariations in a given structure were estimated using structure predictions with permuted HA sequences. Details are provided in Supplementary Methods.

### Cells

MDCK and 293 T cells were cultured as described previously^15^.

### Plasmids

The HA gene segment of A/Anhui/1/05 (H5N1) was cloned into the BsmBI sites of the previously described modified pHW2000 plasmid^64^. The bidirectional plasmids containing the A/PR/8/34 and A/Indonesia/5/05 gene segments were described previously^64^,^65^. Mutations were introduced into the HA gene segment of A/PR/8/34 (H1N1), A/Bilthoven/16190/68 (H3N2) and the HPAI A/Anhui/1/05 (H5N1). Primers to introduce the desired mutations were designed using the web based quick change primer design program (Agilent technologies, Amstelveen, the Netherlands). Mutations were introduced using the QuikChange Multi Site-Directed mutagenesis kit (Agilent) according to the manufacturer’s instructions or using pfu ultra II fusion as described previously^15^.

### Production of recombinant virus

Recombinant viruses were produced using the calcium phosphate method as described previously^15^. Recombinant viruses that contained mutations in the HA gene segment of A/PR/8/34 (H1) or A/Bilthoven/16190/68 (H3) were produced by transfecting 293 T cells with 5 μg of each of the 7 A/PR/8/34 gene segments and 5 μg of the desired mutant or wild-type HA. Viruses containing mutations in the HA segment of A/Anhui/1/05 (H5) were produced with an A/Indonesia/5/05 (H5N1) virus backbone. If no virus was produced after MDCK cells were inoculated with the 293 T supernatant, a second MDCK passage was obtained. If this still did not result in recombinant virus, 1 mU/ml of Vibrio Cholera Neuraminidase (VCNA; Sigma, Zwijndrecht, The Netherlands) and 10 μg/μl L-1-Tosylamide-2-phenylethyl chloromethyl ketone (TPCK) treated trypsin (Sigma) was added the day after transfection. Moreover, both 293 T supernatant and a mixture of 293 T supernatant and resuspended cells were used to inoculate MDCK cells. All recombinant viruses that were used in plaque assays were sequenced using a 3130XL genetic analyser (Applied Biosystems, Bleiswijk, The Netherlands) to check for the presence of the introduced mutations and absence of undesired co-mutations.

### Plaque assay

Virus replication of the mutant viruses was determined using a plaque assay. The plaque assay was performed as described previously^15^. The plaque size was analyzed using ImageQuant TL colony counting software version 8.1 (GE Healthcare, Life sciences, Eindhoven, the Netherlands). Plaque size was plotted as the radius of the plaques (in mm) and displayed using Graphpad Prism 4 (Graphpad Software, La Jolla, USA).

## Additional Information

**How to cite this article**: Gultyaev, A. P. *et al*. Subtype-specific structural constraints in the evolution of influenza A virus hemagglutinin genes. *Sci. Rep.* **6**, 38892; doi: 10.1038/srep38892 (2016).

**Publisher's note:** Springer Nature remains neutral with regard to jurisdictional claims in published maps and institutional affiliations.

## Supplementary Material

Supplementary Information

## Figures and Tables

**Table 1:** Base pairs in predicted local structures with at least one correlation value of 0.8 or more.

nucleotide positions	M(xy)	R_1_(xy)	R_2_(xy)	subtype
180–202	0.46	0.75	0.94	H7
1040–1073	0.31	0.90	0.90	H3N2 human
1587–1593	0.44	0.90	0.65	H13
1549–1555	0.37	0.89	0.87	H8
1222–1234	0.49	0.67	0.89	H10
806–821	0.09	0.88	0.60	H3N2 human
999–1033	0.38	0.88	0.56	H8
1300–1306	0.48	0.87	0.80	H10
1045–1054	0.41	0.86	0.48	H4
178–204	0.41	0.86	0.66	H7
1221–1231	0.41	0.86	0.72	H7
1588–1600	0.37	0.86	0.86	H8
71–82	0.38	0.85	0.85	H13
467–479	0.40	0.73	0.84	H11
1382–1397	0.35	0.83	0.75	H11
801–833	0.03	0.81	0.62	H3N2 human

The pairs are given in descending order of the highest score for a given pair (max[R_1_(xy),R_2_(xy)]). Covariations with perfect scores R_1_(xy) = R_2_(xy) = 1.0 in the H14 and H15 subtypes are not given here because these high values might be determined by small numbers of available HA sequences (16 and 13 strains, respectively) that do not contain mismatches in the putative pairs. These covariations are listed in Supplementary Table S1.

## References

[b1] Horimoto T. & Kawaoka Y. Influenza: Lessons from past pandemics, warnings from current incidents. Nature Rev. Microbiol. 3, 591–600 (2005). 16064053 10.1038/nrmicro1208

[b2] Nelson M. I. & Holmes E. C. The evolution of epidemic influenza. Nature Rev. Genet. 8, 196–205 (2007). 17262054 10.1038/nrg2053

[b3] Ma W., Garcia-Sastre A. & Schwemmle M. Expected and unexpected features of the newly discovered bat influenza A-like viruses. PLoS Pathog. 11, e1004819 (2015). 26042416 10.1371/journal.ppat.1004819 PMC4456350

[b4] Gultyaev A. P., Fouchier R. A. M. & Olsthoorn R. C. L. Influenza virus RNA structure: unique and common features. Int. Rev. Immunol. 29, 533–556 (2010). 20923332 10.3109/08830185.2010.507828

[b5] Gultyaev A. P., Heus H. A. & Olsthoorn R. C. L. An RNA conformational shift in recent H5N1 influenza A viruses. Bioinformatics 23, 272–276 (2007). 17090581 10.1093/bioinformatics/btl559

[b6] Gultyaev A. P. & Olsthoorn R. C. L. A family of non-classical pseudoknots in influenza A and B viruses. RNA Biol. 7, 125–129 (2010). 20200490 10.4161/rna.7.2.11287

[b7] Ilyinskii P. O. . Importance of mRNA secondary structural elements for the expression of influenza virus genes. OMICS 13, 421–430 (2009). 19594376 10.1089/omi.2009.0036

[b8] Moss W. N., Priore S. F. & Turner D. H. Identification of potential conserved RNA secondary structure throughout influenza A coding regions. RNA 17, 991–1011 (2011). 21536710 10.1261/rna.2619511 PMC3096049

[b9] Moss W. N. . The 3′ splice site of influenza A segment 7 mRNA can exist in two conformations: a pseudoknot and a hairpin. PLoS One 7, e38323 (2012). 22685560 10.1371/journal.pone.0038323 PMC3369869

[b10] Priore S. F. . Secondary structure of a conserved domain in the intron of influenza A NS1 mRNA. PLoS One 8, e70615 (2013). 24023714 10.1371/journal.pone.0070615 PMC3759394

[b11] Priore S. F., Kauffmann A. D., Baman J. R. & Turner D. H. The influenza A PB1-F2 and N40 start codons are contained within an RNA pseudoknot. Biochemistry 54, 3413–3415 (2015). 25996464 10.1021/bi501564d PMC4597466

[b12] Jiang T., Kennedy S. D., Moss W. N., Kierzek E. & Turner D. H. Secondary structure of a conserved domain in an intron of influenza A M1 mRNA. Biochemistry 53, 5236–5248 (2014). 25026548 10.1021/bi500611j PMC4139153

[b13] Chen J. L., Kennedy S. D. & Turner D. H. Structural features of a 3′ splice site in influenza A. Biochemistry 54, 3269–3285 (2015). 25909229 10.1021/acs.biochem.5b00012 PMC4455060

[b14] Jiang T., Nogales A., Baker S. F., Martinez-Sobrido L. & Turner D. T. Mutations designed by ensemble defect to misfold conserved RNA structures of influenza A segments 7 and 8 affect splicing and attenuate viral replication in cell culture. PLoS One 11, e0156906 (2016). 27272307 10.1371/journal.pone.0156906 PMC4896458

[b15] Gultyaev A. P. . RNA structural constraints in the evolution of the influenza A virus genome NP segment. RNA Biol. 11, 942–952 (2014). 25180940 10.4161/rna.29730 PMC4179967

[b16] Soszynska-Jozwiak M., Michalak P., Moss W. N., Kierzek R. & Kierzek E. A conserved secondary structural element in the coding region of the influenza A virus nucleoprotein (NP) mRNA is important for the regulation of viral proliferation. PLoS One 10, e0141132 (2015). 26488402 10.1371/journal.pone.0141132 PMC4619443

[b17] Priore S. F., Moss W. N. & Turner D. H. Influenza A virus coding regions exhibit host-specific global ordered RNA structure. PLoS One 7, e35989 (2012). 22558296 10.1371/journal.pone.0035989 PMC3338493

[b18] Baudin F., Bach C., Cusack S. & Ruigrok R. W. H. Structure of influenza virus RNP. I. Influenza virus nucleoprotein melts secondary structure in panhandle RNA and exposes the bases to the solvent. EMBO J. 13, 3158–3165 (1994). 8039508 10.1002/j.1460-2075.1994.tb06614.x PMC395207

[b19] Moeller A., Kirchdoerfer R. N., Potter C. S., Carragher B. & Wilson I. A. Organization of the influenza virus replication machinery. Science 338, 1631–1634 (2012). 23180774 10.1126/science.1227270 PMC3578580

[b20] Arranz R. . The structure of native influenza virion ribonucleoproteins. Science 338, 1634–1637 (2012). 23180776 10.1126/science.1228172

[b21] Lenartowicz E. . Self-folding of naked segment 8 genomic RNA of influenza A virus. PLOS One 11, e0148281 (2016). 26848969 10.1371/journal.pone.0148281 PMC4743857

[b22] Noda T. . Three-dimensional analysis of ribonucleoprotein complexes in influenza A virus. Nature Commun. 3, 639 (2012). 22273677 10.1038/ncomms1647 PMC3272569

[b23] Fournier E. . A supramolecular assembly formed by influenza A virus genomic RNA segments. Nucleic Acids Res. 40, 2197–2209 (2012). 22075989 10.1093/nar/gkr985 PMC3300030

[b24] Gavazzi C. . An *in vitro* network of intermolecular interactions between viral RNA segments of an avian H5N2 influenza A virus: comparison with a human H3N2 virus. Nucleic Acids Res. 41, 1241–1254 (2013). 23221636 10.1093/nar/gks1181 PMC3553942

[b25] Gavazzi C. . A functional sequence-specific interaction between influenza A virus genomic RNA segments. Proc. Natl. Acad. Sci. USA 110, 16604–16609 (2013). 24067651 10.1073/pnas.1314419110 PMC3799358

[b26] Gerber M., Isel C., Moules V. & Marquet R. Selective packaging of the influenza A genome and consequences for genetic reassortment. Trends Microbiol. 22, 446–455 (2014). 24798745 10.1016/j.tim.2014.04.001

[b27] Hutchinson E. C., von Kirchbach J. C., Gog J. R. & Digard P. Genome packaging in influenza A virus. J. Gen. Virol. 9, 313–328 (2010). 10.1099/vir.0.017608-0 19955561

[b28] Yamanaka K., Ishihama A. & Nagata K. Reconstitution of influenza virus RNA-nucleoprotein complexes structurally resembling native viral ribonucleoprotein cores. J. Biol. Chem. 265, 11151–11155 (1990). 2358455

[b29] Gog J. R. . Codon conservation in the influenza A virus genome defines RNA packaging signals. Nucleic Acids Res. 35, 1897–1907 (2007). 17332012 10.1093/nar/gkm087 PMC1874621

[b30] Essere B. . Critical role of segment-specific packaging signals in genetic reassortment of influenza A viruses. Proc. Natl. Acad. Sci. USA 110, E3840–E3848 (2013). 24043788 10.1073/pnas.1308649110 PMC3791739

[b31] Lubeck M. D., Palese P. & Schulman J. L. Nonrandom association of parental genes in influenza A virus recombinants. Virology 95, 269–274 (1979). 442543 10.1016/0042-6822(79)90430-6

[b32] Schrauwen E. J. . Reassortment between avian H5N1 and human influenza viruses is mainly restricted to the matrix and neuraminidase gene segments. PLoS One 8, e59889 (2013). 23527283 10.1371/journal.pone.0059889 PMC3604002

[b33] Bernhart S. H., Hofacker I. L., Will S., Gruber A. R. & Stadler P. F. RNAalifold: improved consensus structure prediction for RNA alignments. BMC Bioinformatics 9, 474 (2008). 19014431 10.1186/1471-2105-9-474 PMC2621365

[b34] Gruber A. R., Findeiß S., Washietl S., Hofacker I. L. & Stadler P. F. RNAz 2.0:improved noncoding RNA detection. Pac. Symp. Biocomput. 2010, 69–79 (2010). 19908359

[b35] Smith D. J. . Mapping the antigenic and genetic evolution of influenza virus. Science 305, 371–376 (2004). 15218094 10.1126/science.1097211

[b36] Koel B. F. . Substitutions near the receptor binding site determine major antigenic change during influenza virus evolution. Science 342, 976–979 (2013). 24264991 10.1126/science.1244730

[b37] Klenk H. D. & Garten W. Host cell proteases controlling virus pathogenicity. Trends Microbiol. 2, 39–43 (1994). 8162439 10.1016/0966-842x(94)90123-6

[b38] Dugan V. G. . The evolutionary genetics and emergence of avian influenza viruses in wild birds. PLoS Pathog. 4, e1000076 (2008). 18516303 10.1371/journal.ppat.1000076 PMC2387073

[b39] Liu S. . Panorama phylogenetic diversity and distribution of type A influenza virus. PLoS One 4, e5022 (2009). 19325912 10.1371/journal.pone.0005022 PMC2658884

[b40] Zuker M. Mfold web server for nucleic acid folding and hybridization prediction. Nucleic Acids Res. 31, 3406–3415 (2003). 12824337 10.1093/nar/gkg595 PMC169194

[b41] Smith G. J. D. . Nomenclature updates resulting from the evolution of avian influenza A(H5) virus clades 2.1.3.2a, 2.2.1, and 2.3.4 during 2013-2014. Influenza Other Respir. Viruses 9, 271–276 (2015). 25966311 10.1111/irv.12324 PMC4548997

[b42] Duan L. . Characterization of low-pathogenic H5 subtype influenza viruses from Eurasia: implications for the origin of highly pathogenic H5N1 viruses. J. Virol. 81, 7529–7539 (2007). 17507485 10.1128/JVI.00327-07 PMC1933357

[b43] Lupiani B. & Reddy S. M. The history of avian influenza. Comp. Immun. Microbiol. Infect. Dis. 32, 311–323 (2009). 10.1016/j.cimid.2008.01.004 18533261

[b44] Marsh G. A., Hatami R. & Palese P. Specific residues of the influenza A virus hemagglutinin viral RNA are important for efficient packaging into budding virions. J. Virol. 81, 9727–9736 (2007). 17634232 10.1128/JVI.01144-07 PMC2045411

[b45] Wong E. H. M., Smith D. K., Rabadan R., Peiris M. & Poon L. L. M. Codon usage bias and the evolution of influenza A viruses. Codon usage biases of influenza virus. BMC Evol. Biol. 10, 253 (2010). 20723216 10.1186/1471-2148-10-253 PMC2933640

[b46] Biologics Corporation. Codon Adaptation Index Calculator. Available at: www.biologicscorp.com. (Accessed: 25th October 2016).

[b47] Coleman J. R. . Virus attenuation by genome-scale changes in codon pair bias. Science 320, 1785–1787 (2008). 10.1126/science.1155761 PMC2754401 18583614

[b48] Mueller S. . Live attenuated influenza virus vaccines by computer-aided rational design. Nature Biotechnol. 28, 723–726 (2010). 20543832 10.1038/nbt.1636 PMC2902615

[b49] Fan R. L. Y. . Generation of live attenuated influenza virus by using codon usage bias. J. Virol. 89, 10762–10773 (2015). 26269186 10.1128/JVI.01443-15 PMC4621104

[b50] Akmaev V. R., Kelley S. T. & Stormo G. D. Phylogenetically enhanced statistical tools for RNA structure prediction. Bioinformatics 16, 501–512 (2000). 10980147 10.1093/bioinformatics/16.6.501

[b51] Dutheil J. Y. Detecting coevolving positions in a molecule: why an how to account for phylogeny. Brief. Bioinformatics 13, 228–243 (2012). 21949241 10.1093/bib/bbr048

[b52] Talavera D., Lovell S. C. & Whelan S. Covariation is a poor measure of molecular coevolution. Mol. Biol. Evol. 32, 2456–2468 (2015). 25944916 10.1093/molbev/msv109 PMC4540965

[b53] Gutell R. R., Power A., Hertz G. Z., Putz E. J. & Stormo G. D. Identifying constraints on the higher-order structure of RNA: continued development and application of comparative sequence analysis methods. Nucleic Acids Res. 20, 5785–5795 (1992). 1454539 10.1093/nar/20.21.5785 PMC334417

[b54] Shang L., Xu W., Ozer S. & Gutell R. R. Structural constraints identified with covariation analysis in ribosomal RNA. PLoS One 7, e39383 (2012). 22724009 10.1371/journal.pone.0039383 PMC3378556

[b55] Mortimer S. A., Kidwell M. A. & Doudna J. A. Insights into RNA structure and function from genome-wide studies. Nature Rev. Genet. 15, 469–479 (2014). 24821474 10.1038/nrg3681

[b56] Knoepfel S. A. & Berkhout B. On the role of four small hairpins in the HIV-1 RNA genome. RNA Biol. 10, 540–552 (2013). 23535706 10.4161/rna.24133 PMC3710360

[b57] McFadden N. . Influence of genome-scale RNA structure disruption on the replication of murine norovirus - similar replication kinetics in cell culture but attenuation of viral fitness *in vivo*. Nucleic Acids Res. 41, 6316–6331 (2013). 23630317 10.1093/nar/gkt334 PMC3695492

[b58] Garcia M., Crawford J. M., Latimer J. W., Rivera-Cruz E. & Perdue M. L. Heterogeneity in the haemagglutinin gene and emergence of the highly pathogenic phenotype among recent H5N2 avian influenza viruses from Mexico. J. Gen. Virol. 77, 1493–1504 (1996). 8757992 10.1099/0022-1317-77-7-1493

[b59] Perdue M. L., Garcia M., Senne D. & Fraire M. Virulence-associated sequence duplication at the hemagglutinin cleavage site of avian influenza viruses. Virus Res. 49, 173–186 (1997). 9213392 10.1016/s0168-1702(97)01468-8

[b60] Simon-Loriere E., Martin D. P., Weeks K. M. & Negroni M. RNA structures facilitate recombination-mediated gene swapping in HIV-1. J. Virol. 84, 12675–12682 (2010). 20881047 10.1128/JVI.01302-10 PMC3004330

[b61] Penno C. . Productive mRNA stem loop-mediated transcriptional slippage: crucial features in common with intrinsic terminators. Proc. Natl. Acad. Sci. USA 112, E1984–E1993 (2015). 25848054 10.1073/pnas.1418384112 PMC4413344

[b62] Mehedi M. . Ebola virus RNA editing depends on the primary editing site sequence and an upstream secondary structure. PLoS Pathog. 9, e1003677 (2013). 24146620 10.1371/journal.ppat.1003677 PMC3798607

[b63] Fujii K., Ozawa M., Iwatsuki-Horimoto K., Horimoto T. & Kawaoka Y. Incorporation of influenza A virus genome segments does not absolutely require wild-type sequences. J. Gen. Virol. 90, 1734–1740 (2009). 19297607 10.1099/vir.0.010355-0 PMC2731938

[b64] de Wit E. . Efficient generation and growth of influenza virus A/PR/8/34 from eight cDNA fragments. Virus Res. 103, 155–161 (2004). 15163504 10.1016/j.virusres.2004.02.028

[b65] Chutinimitkul S. . *In vitro* assessment of attachment pattern and replication efficiency of H5N1 influenza A viruses with altered receptor specificity. J. Virol. 84, 6825–6833 (2010). 20392847 10.1128/JVI.02737-09 PMC2903244

