# Supplementary material for: Subtype-specific structural constraints in the evolution of influenza A virus hemagglutinin genes
Source: Sci Rep. 2016 Dec 14;6:38892. doi: 10.1038/srep38892 (PMC5155281; doi:10.1038/srep38892)
Supplement: Supplementary Information [file srep38892-s1.pdf]

SUPPLEMENTARY INFORMATION

Subtype-specific structural constraints in the evolution of influenza A virus hemagglutinin genes.

Alexander P. Gultyaev, Monique I. Spronken, Mathilde Richard, Eefje J.A. Schrauwen, Rene C. L. Olsthoorn & Ron A.M. Fouchier

SUPPLEMENTARY METHODS

RNA structure predictions. Predictions of conserved RNA structures by RNAalifold[1] and RNAz[2] were carried out on datasets containing HA sequences representative for a given subtype. For each of the HA subtypes three datasets of representative virus strains were constructed. Within the datasets, the representatives were selected from different clusters with an attempt to have as much sequence variability as possible. For this purpose, published HA phylogenetic trees[3-19] and neighbor-joining trees generated using The Influenza Virus Resource[20] were used. Each of the datasets for HA subtypes with only avian strains contained six representatives, with three sequences of an Eurasian origin and three American strains, as this is usually the main speciation line[3]. It was not possible to derive a single similarity threshold for sequences of all subtypes due to different intra-subtype diversity levels, but construction of datasets containing sequences from the avian strains from different hemispheres maintained the diversity at maximum levels possible for each subtype, with an average level of about 8%[3]. For subtypes H1, H2 and H3, which include human and swine virus lineages, both subtype-specific and host-specific triplets of datasets were constructed. Host-specific datasets contained 6 or 5 sequences corresponding to the main sublineages in these HA clades. Some representative sequences were used in both subtype-specific and host-specific dataset triplets. In the H14 and H15 dataset triplets, some sequences were used more than once in different combinations due to the lack of available sequences. Such over-representation did not result in a significant bias in predictions due to overall high similarities between available sequences of these subtypes, and alternative dataset constructions with different over-represented sequences did not change the predictions.

The HA-like segments of the influenza A-like bat viruses (three H17 and one H18 sequence available) were not considered. The datasets with representative strains are listed below.

<u>Subtype H1</u>: A/mallard/Alberta/35/1976(H1N1), accession D10477; A/duck/NZL/160/1976(H1N3), CY005735; A/Puerto Rico/8/34(H1N1), EF467821; A/New Caledonia/20/1999(H1N1), CY031336; A/swine/St-Hyacinthe/148/1990(H1N1), U11703; A/Iowa/CEID23/2005(H1N1), DQ889689 (dataset H1-1); A/duck/Miyagi/66/1977(H1N1), AB271113; A/Swine/Spain/50047/2003(H1N1), CY009892; A/South Carolina/1/18 (H1N1), AF117241; A/Memphis/7/1980(H1N1), CY010908; A/swine/Iowa/15/30 (H1N1), AF091308; A/California/04/2009(H1N1), FJ966082 (dataset H1-2);

A/swine/Netherlands/3/80(H1N1), AF091314; A/pintail/Ohio/25/1999(H1N1), CY017725; A/Bel/1942(H1N1), CY009276; A/Washington/10/2008(H1N1), FJ686964; A/swine/USA/1976/1931(H1N1), CY045740; A/swine/Wisconsin/1/1957(H1N1), CY026283 (dataset H1-3);

A/mallard/Alberta/127/1977(H1N1), CY004592; A/mallard duck/New York/170/1982(H1N2), CY014901; A/mallard/ALB/201/1998(H1N1), CY004507; A/duck/NZL/160/1976(H1N3), CY005735; A/mallard/Sweden/3/2002(H1N2), CY060268; A/pintail/Aomori/422/2007(H1N1), AB546149 (dataset H1avian-1);

A/murre/Alaska/305/1976(H1N6), CY015163; A/pintail duck/ALB/238/1979(H1N1), CY004482; A/blue-winged teal/Ohio/907/2002(H1N6), CY020861; A/duck/Miyagi/66/1977(H1N1), AB271113; A/duck/Italy/69238/2007(H1N1), FJ432754; A/duck/Zhejiang/0607-13/2011(H1N2), JN605373 (dataset H1avian-2);

A/pintail/Ohio/25/1999(H1N1), CY017725; A/mallard/Alberta/42/1977(H1N6), CY004458; A/pintail duck/Alberta/210/2002(H1N1) CY004546; A/duck/Australia/749/1980(H1N1) CY014627; A/duck/HK/196/1977(H1N1), D00839; A/Bewick's swan/Netherlands/1/2007(H1N5), CY076976 (dataset H1avian-3);

A/South Carolina/1/1918(H1N1), AF117241; A/USSR/90/1977(H1N1), CY010372; A/Texas/36/1991(H1N1), AY289927; A/New Caledonia/20/1999(H1N1), CY031336; A/Wisconsin/13/2009(H1N1), GQ476111 (dataset H1human-1);

A/Puerto Rico/8/1934(H1N1), EF467821; A/Chile/1/1983(H1N1), CY121261; A/Shengzhen/227/1995(H1N1), CY125052; A/Denmark/3/2005(H1N1), EU097950; A/Solomon Islands/3/2006(H1N1), EU124177 (dataset H1human-2);

A/India/6263/1980(H1N1), CY020453; A/Singapore/6/1986(H1N1), CY020477; A/Beijing/262/1995(H1N1), AY289928; A/Pennsylvania/01/2007(H1N1), EU199290; A/Brisbane/59/2007(H1N1), CY030232 (dataset H1human-3);

A/swine/St-Hyacinthe/148/1990(H1N1), U11703; A/swine/Wisconsin/1/1961(H1N1), AF091307; A/swine/Miyagi/5/2003(H1N2), AB294217; A/Iowa/CEID23/2005(H1N1), DQ889689; A/swine/OH/511445/2007(H1N1), EU604689 (dataset H1swine-1);

A/swine/Iowa/15/30(H1N1), AF091308; A/swine/Tennessee/10/1978(H1N1), CY024986; A/swine/Taiwan/CO935/2004(H1N2), DQ447187; A/swine/Wisconsin/238/97(H1N1), AF222033; A/California/04/2009(H1N1), FJ966082 (dataset H1swine-2);

A/swine/USA/1976/1931(H1N1), CY045740; A/swine/Wisconsin/1/1957(H1N1), CY026283; A/swine/Thailand/HF6/2005(H1N1), FJ688266; A/swine/Zhejiang/1/2004(H1N2), DQ139320; A/swine/Indiana/P12439/00(H1N2), AF455680 (dataset H1swine-3).

Subtype H2: A/gull/MD/19/1977(H2N9), CY005808; A/mallard/Alberta/149/2002(H2N4), CY003984; A/laughing gull/NJ/75/1985(H2N9), CY003863; A/duck/GDR/72 (H2N9), L11129; A/herring gull/Delaware/670/1988(H2N9), CY014556; A/Berkeley/1/68 (H2N2), L11125 (dataset H2-1);

A/mallard/Ontario/56/76 (H2N3), L11138; A/chicken/New York/13828-3/1995(H2N2), CY014821; A/mallard/Alberta/77/1977(H2N3), CY003847; A/Japan/305/1957(H2N2), CY014976; A/mallard/Potsdam/177-4/1983(H2N2), CY005765; A/duck/Nanchang/2-0486/2000(H2N9), CY014608 (dataset H2-2);

A/sanderling/NJ/766/1986(H2N7), CY003887; A/semi-palmated sandpiper/Brazil/43/1990(H2N1), CY005413; A/teal/Alberta/16/97(H2N9), AY633388; A/mallard/Netherlands/13/99(H2N2), AY684893; A/Pintail/Praimoric/625/76 (H2N2), L11141; A/Berlin/3/64 (H2N2), L11126 (dataset H2-3);

A/gull/MD/19/1977(H2N9), CY005808; A/mallard/Alberta/149/2002(H2N4), CY003984; A/laughing gull/NJ/75/1985(H2N9), CY003863; A/duck/GDR/72 (H2N9), L11129; A/herring gull/Delaware/670/1988(H2N9), CY014556; A/duck/Hong Kong/273/1978(H2N2), L11128 (dataset H2avian-1);

A/mallard/Ontario/56/76 (H2N3), L11138; A/chicken/New York/13828-3/1995(H2N2), CY014821; A/mallard/Alberta/77/1977(H2N3), CY003847; A/ruddy turnstone/Delaware/34/1993(H2N1), CY015135; A/mallard/Potsdam/177-4/1983(H2N2), CY005765; A/duck/Nanchang/2-0486/2000(H2N9), CY014608 (dataset H2-2);

A/sanderling/NJ/766/1986(H2N7), CY003887; A/semi-palmated sandpiper/Brazil/43/1990(H2N1), CY005413; A/teal/Alberta/16/97(H2N9), AY633388; A/mallard/Netherlands/13/99(H2N2), AY684893; A/Pintail/Praimoric/625/76 (H2N2), L11141; A/mallard/MT/Y61(H2N2), CY116843 (dataset H2avian-3);

A/Japan/305/1957(H2N2), CY014976; A/England/1/1961(H2N2), CY125854; A/Taiwan/1964(H2N2), DQ508881; A/Johannesburg/617/1967(H2N2), CY032285; A/Ann Arbor/7/1967(H2N2), CY125838 (dataset H2human-1);

A/Singapore/1/1957(H2N2), L20410; A/Ned/65/1963(H2N2), CY125886; A/Berlin/3/1964(H2N2), L11126; A/Montevideo/2208/1967(H2N2), CY125822; A/Tashkent/1046/1967(H2N2), CY032277 (dataset H2human-2); A/Albany/22/1957(H2N2), CY021805; A/Netherlands/056H1/1960(H2N2), CY077786; A/Moscow/1019/1965(H2N2), CY031603; A/Georgia/1/1967(H2N2), CY033980; A/Berkeley/1/1968(H2N2), L11125 (dataset H2human-3).

Subtype H3: A/duck/Hong Kong/7/1975(H3N2), CY006026; A/mallard/Ohio/181/1986(H3N1), CY021429; A/mallard duck/New York/157/1986(H3N6), CY014865; A/Aichi/2/68(H3N2), V01085; A/Moscow/10/99(H3N2), DQ487341; A/Guangdong/423/2009(H3N2), CY091839 (dataset H3-1);

A/mallard/Netherlands/2/1999(H3N5), CY060261; A/duck/Korea/JS53/2004(H3N2), JN087096; A/pintail duck/ALB/462/1979(H3N6), CY005938; A/Udorn/307/1972(H3N2), M54895; A/Netherlands/241/1993(H3N2), CY112613; A/HaNoi/N080/2007(H3N2), CY105606 (dataset H3-2);

A/common teal/Sweden/1/2003(H3N3), CY060185; A/mallard duck/ALB/676/1979(H3N6), CY005916; A/mallard/Ohio/1801/2005(H3N8), CY021341; A/Sichuan/2/1987(H3N2), CY121293; A/Vienna/47/96M(H3N2), AF017270; A/Guangdong/17/2007(H3N2), CY091831 (dataset H3-3);

A/duck/Hong Kong/7/1975(H3N2), CY006026; A/duck/Hunan/S1824/2012(H3N8), CY146628; A/duck/Korea/U5-2/2007(H3N8), JN087144;A/mallard/Ohio/181/1986(H3N1), CY021429; A/mallard duck/New York/157/1986(H3N6), CY014865; A/mallard/Maryland/691/2005(H3N2), CY021269 (dataset H3avian-1);

A/mallard/Netherlands/2/1999(H3N5), CY060261; A/duck/Korea/JS53/2004(H3N2), JN087096; A/duck/Nanchang/1681/1992(H3N8), CY006016; A/pintail duck/ALB/462/1979(H3N6), CY005938; A/pintail/Alaska/49/2005(H3N8), CY020877; A/longtail duck/Maryland/291/2005(H3N8), CY017773 (dataset H3avian-2);

A/duck/Hokkaido/10/1985(H3N8), AB276113; A/chicken/Vietnam/G14/2008(H3N8), AB593455; A/common teal/Sweden/1/2003(H3N3), CY060185; A/mallard duck/ALB/676/1979(H3N6), CY005916; A/pintail/Alaska/779/2005(H3N8), CY017757; A/mallard/Ohio/1801/2005(H3N8), CY021341 (dataset H3avian-3);

A/Aichi/2/68(H3N2), V01085; A/England/321/1977(H3N2), X05907; A/Houston/56798/1992(H3N2), CY113693; A/Moscow/10/99(H3N2), DQ487341; A/Vienna/28/2006(H3N2), JF340085; A/Guangdong/423/2009(H3N2), CY091839 (dataset H3human-1);

A/Udorn/307/1972(H3N2), M54895; A/Netherlands/233/1982(H3N2), CY077834; A/Netherlands/241/1993(H3N2), CY112613; A/Netherlands/213/2003(H3N2), HQ166052; A/HaNoi/N080/2007(H3N2), CY105606 (dataset H3human-2);

A/Victoria/3/1975(H3N2), V01098; A/Sichuan/2/1987(H3N2), CY121293; A/Vienna/47/96M(H3N2), AF017270; A/reassortant/CDC2005712034(California/07/2004 x Puerto Rico/8/1934)(H3N2), JF690267; A/Guangdong/17/2007(H3N2), CY091831 (dataset H3human-3).

Subtype H4: A/duck/Czeckoslovakia/1956(N4N6), M25283; A/duck/Hong Kong/24/1976(H4N2), CY006030; A/gray teal/AUS/3/1979(H4N6), CY005679; A/mallard duck/ALB/581/1983(H4N4), CY005957; A/mallard duck/New York/180/1986(H4N9), CY014857; A/mallard/ALB/49/1995(H4N6), CY004911 (dataset H4-1); A/red-necked stint/Australia/4189/1980(H4N8), CY014630; A/Duck/Nanchang/4-165/2000(H4N6), CY006017; A/duck/Hong Kong/365/1978(H4N6), CY006027; A/chicken/Alabama/1/1975(H4N8), M25288; A/pintail/Alberta/207/1999(H4N8), CY004933; A/turkey/Minnesota/833/1980(H4N2), M25290 (dataset H4-2); A/duck/New Zealand/31/1976(H4N6), M25286; A/duck/Hokkaido/1058/2001(H4N5), AB288842; A/duck/Hong Kong/229/1977(H4N3), AB292408; A/ruddy turnstone/NJ/47/1985(H4N6), CY005958; A/pintail/Alberta/269/2001(H4N6), CY005966; A/mallard/Ohio/655/2002(H4N6), CY020773 (dataset H4-3).

Subtype H5: A/duck/France/02166/2002(H5N3), AJ632268; A/mallard/Netherlands/3/1999(H5N2), AY684894; A/goose/Guangdong/1/96(H5N1), AF148678; A/mallard/MN/133/1998(H5N2), EF607872; A/turkey/Wisconsin/1/1968(H5N9), CY080507; A/shorebird/DE/1346/2001(H5N7), EF607889 (dataset H5-1); A/chicken/Italy/9097/97(H5N9), AF194992; A/mallard/Bavaria/1/2005(H5N2), DQ387854; A/duck/Singapore/3/1997(H5N3), EF619972; A/duck/Minnesota/1525/1981(H5N1), CY014726; A/ruddy turnstone/New Jersey/2242/2000(H5N3), AY296084; A/chicken/Hidalgo/28159-232/1994(H5N2), CY006040 (dataset H5-2); A/duck/Hong Kong/312/1978(H5N3), EF597248; A/mallard/Italy/36/2002(H5N3), EF597268; A/Anhui/1/2007(H5N1), CY098681; A/chicken/TX/298313/2004(H5N2), AY849793; A/blue goose/WI/711/1975(H5N2), EF607857; A/chicken/Pennsylvania/1/1983(H5N2), CY015073 (dataset H5-3).

Subtype H6: A/mallard/Netherlands/16/99(H6N5), AY684892; A/Teal/Hong Kong/W312/97(H6N1), AF250479; A/duck/Hong Kong/221/77 (H6N8), AJ410545; A/sanderling/Delaware/1258/1986(H6N6), CY014607; A/pintail/Ohio/226/1998(H6N2), CY013255; A/mallard/Alberta/4/1994(H6N8), CY127072 (dataset H6-1);

A/teal/Norway/10_476/2005(H6N2), FM179757; A/duck/Shantou/339/2000(H6N2), HM144398; A/duck/Hong Kong/323/98(H6N2), AJ410529; A/mallard duck/ALB/250/1978(H6N2), CY004034; A/mallard/Ohio/170/1999(H6N5), CY015484; A/mallard duck/ALB/10/1985(H6N2), CY004186 (dataset H6-2);

A/chicken/Taiwan/0204/05(H6N1), DQ376652; A/wild duck/Shantou/2853/2003(H6N2), HM144472; A/mallard/Sweden/81/2002(H6N1), CY060382; A/pintail duck/ALB/1040/1979(H6N5), CY004072; A/mallard/Maryland/887/2002(H6N1), EU026007; A/mallard duck/ALB/155/1990(H6N3), CY004226 (dataset H6-3).

Subtype H7: A/turkey/England/647/77(H7N7), AF202247; A/Mallard/Sweden/56/02(H7N7), AY999977; A/chicken/Netherlands/1/03(H7N7), AY338458; A/chicken/Chile/176822/02(H7N3), AY303630; A/chicken/New York/12273-11/1999(H7N3), AY240892; A/northern shoveler/California/HKWF1026/2007(H7N3), CY039580 (dataset H7-1);

A/FPV/Rostock/1934(H7N1), M24457; A/duck/Hokkaido/143/2003(H7N1), AB269694; A/quail/Italy/3347/2004(H7N3), CY020613; A/mallard duck/ALB/224/1977(H7N5), CY005973; A/mallard duck/Alberta/435/1985(H7N3), CY014587; A/Canada/rv504/2004(H7N3), CY015006 (dataset H7-2);

A/chicken/Pakistan/447/95(H7N3), AF202226; A/Chicken/Italy/1067/99(H7N1), AJ584647; A/mute swan/Hungary/5973/2007(H7N7), GQ240813; A/turkey/Minnesota/1200/1980(H7N3), CY014778; A/black duck/Maryland/415/2001(H7N3), CY020885; A/chicken/New York/19499/2005(H7N2), CY034246 (dataset H7-3).

Subtype H8: A/turkey/Ontario/6118/1968(H8N4), CY014659; A/mallard/Alaska/708/2005(H8N4), CY017749; A/northern shoveler/California/3676/2010(H8N2), CY134295; A/mallard/Netherlands/1/2006(H8N4), CY043848; A/teal/Chany/444/2009(H8N8), CY098524; A/mallard/Sweden/8/2003(H8N4), CY060404 (dataset H8-1);

A/mallard/Alberta/283/1977(H8N4), CY005970; A/mallard/ALB/194/1992(H8N4), CY005972; A/northern shoveler/California/HKWF1325/2007(H8N4), CY034159; A/duck/Hokkaido/95/1981(H8N4), AB450454; A/Anas crecca/Spain/1459/2008(H8N4), FN386466; A/duck/Tsukuba/255/2005(H8N5), AB669137 (dataset H8-2);

A/mallard duck/Alberta/7/1987(H8N4), CY014583; A/duck/Alaska/702/1991(H8N2), CY015173; A/blue-winged teal/Guatemala/CIP049-07/2010(H8N4), CY096696; A/duck/Thailand/SP-355/2007(H8N4), FJ802406; A/mallard/Sweden/24/2002(H8N4), CY060249; A/common teal/Netherlands/1/2005(H8N4), CY041258 (dataset H8-3).

Subtype H9: A/turkey/Wisconsin/66(H9N2), CY014663; A/rosy-billed pochard/Argentina/CIP051-559/2007(H9N2), CY111590; A/duck/Hong Kong/702/1979(H9N2), CY031259; A/goose/MN/5733-1/1980(H9N2), CY006042; A/quail/Hong Kong/G1/97(H9N2), AF156378; A/chicken/Beijing/1/94(H9N2), AF156380 (dataset H9-1);

A/turkey/California/189/66(H9N2), AF156390; A/turkey/Minnesota/38391-6/95(H9N2), AF156387; A/duck/NZL/76/1984(H9N1), CY005746; A/mallard/Alberta/11/1991(H9N2), CY005990; A/turkey/Netherlands/11015452/2011(H9N2), JX273570; A/chicken/Israel/786/2001(H9N2), EF492231 (dataset H9-2);

A/chicken/Heilongjiang/35/00(H9N2), DQ064366; A/shorebird/Delaware Bay/260/1996(H9N9), CY101224; A/duck/Hong Kong/366/78(H9N2), AY206674; A/knot/DE/2552/1987(H9N5), CY005929; A/duck/Germany/113/1995(H9N2), HE802066; A/chicken/Pakistan/2/1999(H9N2), KF188299 (dataset H9-3).

Subtype H10: A/chicken/Germany/N/1949(H10N7), CY014671; A/swan/Shimane/1331/1981(H10N6), AB289339; A/mallard/Switzerland/WV1090023/2009(H10), HM179251; A/blue-winged teal/ALB/778/1978(H10N3), CY005994; A/red knot/Delaware/1269/2000(H10N7), GU050906; A/mallard/Alberta/209/2003(H10N7), CY005922 (dataset H10-1);

A/quail/Italy/1117/1965(H10N8), CY014644; A/fowl/Hampshire/PD378/1985(H10N4), GQ176120; A/duck/Hokkaido/W87/2007(H10N2), AB450443; A/mallard/Ohio/122/1989(H10N7), CY020925; A/pintail/Alberta/202/2000(H10N7), CY005999; A/northern shoveler/California/HKWF592/2007(H10N7), CY034180 (dataset H10-2);

A/duck/Hong Kong/938/80(H10N1), AB271117; A/duck/Hong Kong/562/1979(H10N9), CY014619; A/chicken/Jiangsu/RD5/2013(H10N9), KF006414; A/green-winged teal/LA/169GW/1988(H10N7), EU743314; A/ruddy turnstone/NJ/1600/2001(H10N7), EU743418; A/American green-winged teal/Ohio/13OS1869/2013(H10N8), KJ568017 (dataset H10-3).

Subtype H11: A/shoveler/Netherlands/19/1999(H11N9), CY014719; A/mallard/Sweden/25/2002(H11N3), CY060254; A/duck/England/1/1956(H11N6), CY130062; A/mallard/ALB/124/1991(H11N2), CY006003; A/mallard duck/ALB/797/1983(H11N3), CY006002; A/environment/Delaware/232/2005(H11N8), CY021149 (dataset H11-1);

A/mallard/Netherlands/7/99(H11N2), AY684895; A/swan/Shimane/48/1997(H11N2), AB277756; A/spotbill duck/Xuyi/6/2005(H11N2), GQ184327; A/duck/Washington/663/1997(H11N9), EF599120; A/ruddy turnstone/Delaware/2762/1987(H11N2), CY014595; A/semipalmated sandpiper/Delaware/2109/2000(H11N6), GU051072 (dataset H11-2);

A/duck/Bavaria/49/2006(H11N1), GU046752; A/duck/Miyagi/47/1977(H11N1), AB450451; A/Baikal teal/Hongze/14/2005(H11N9), GQ184329; A/pintail/Alberta/84/2000(H11N9), CY006004; A/mallard/Minnesota/249/2000(H11N9), GQ257373; A/duck/Memphis/546/1974(H11N9), CY014687 (dataset H11-3).

Subtype H12: A/duck/Alberta/60/1976(H12N5), CY130078; A/pintail/Alberta/49/2003(H12N5), CY005920; A/ruddy turnstone/Delaware/AI03-378/2003(H12N4), CY144381; A/red-necked stint/Australia/5745/1981(H12N9), CY014636; A/mallard/Netherlands/20/2005(H12N8), CY076968; A/duck/Tsukuba/212/2006(H12N5), AB669140 (dataset H12-1);

A/mallard duck/Alberta/342/1983(H12N1), CY006006; A/mallard/Maryland/1135/2005(H12N5), CY021293; A/pintail/Alaska/102/2005(H12N5), CY017733; A/duck/Hokkaido/W26/2012(H12N1), AB780369; A/mallard/Sweden/100103/2009(H12N5), JX566034; A/whooper swan/Mongolia/232/2005(H12N3), GQ907350 (dataset H12-2);

A/mallard/Ohio/409/1988(H12N5), CY016419; A/mallard/North Dakota/Sg-00703/2008(H12N4), CY042954; A/mallard/ALB/52/1997(H12N5), CY005925; A/wild goose/Dongting/C1037/2011(H12N8), KC876691; A/teal/Norway/10_1836/2006(H12N2), FM179754; A/duck/Hokkaido/66/01(H12N5), AB288843 (dataset H12-3).

Subtype H13: A/black-headed gull/Sweden/1/1999(H13N6), AY684887; A/glaucous gull/Alaska/44199-097/2006(H13N3), HM059995; A/herring gull/Delaware/660/1988(H13N6), CY014603; A/gull/Maryland/704/1977 (H13N6), CY014694; A/duck/Siberia/272/1998(H13N6), AB284988; A/Mongolian gull/Mongolia/405/2007(H13N6), GQ907318 (dataset H13-1);

A/black-headed gull/Astrakhan/227/84(H13N6), M26089; A/American white pelican/Minnesota/AI-07-1819/2007 (H13N9), CY054300; A/herring gull/NJ/782/1986(H13N2), CY005932; A/kelp gull/Argentina/LDC4/2006(H13N9), EU523136; A/glaucous-winged gull/SC Alaska/9JR0747R0/2009(H13N6), CY070866; A/great black-headed gull/Atyrau/743/2004(H13N6), GU982281 (dataset H13-2);

A/shorebird/DE/68/2004(H13N9), CY005931; A/gull/Minnesota/945/1980(H13N6), CY014720; A/herring gull/DE/475/1986(H13N2), CY005914; A/gull/Astrakhan/226/1984(H13N6), EU835895; A/black-headed gull/Republic of Georgia/7/2011(H13N6), CY185489; A/herring gull/Norway/10_2336/2006(H13N6), FM179758 (dataset H13-3).

Subtype H14: A/mallard/Astrakhan/263/1982(H14N5), CY014604; A/Gurjev/244/1982(H14N6), M35996; A/herring gull/Astrakhan/267/1982(H14N5), FJ975075 (datasets H14-1, H14-2, H14-3);

A/white-winged scoter/Wisconsin/10OS3922/2010(mixed), JN696315; A/northern shoveler/Missouri/10OS4673/2010(H14N6), CY133381; A/northern shoveler/Mississippi/12OS456/2012(H14N2), CY167267 (dataset H14-1);

A/long-tailed duck/Wisconsin/10OS3912/2010(H14N6), JN696314; A/blue-winged teal/Guatemala/CIP049H106-62/2011(H14N6), KJ195668; A/northern shoveler/California/2696/2011(H14N2), CY146897 (dataset H14-2);

A/long-tailed duck/Wisconsin/10OS4225/2010(H14N6), JN696316; A/blue-winged teal/Guatemala/CIP049H105-15/2011 (H14N3), KJ195676; A/blue-winged teal/TX/AI13-1028/2013(H14N5), KF986854 (dataset H14-3).

Subtype H15: A/teal/Chany/7119/2008(H15N4), CY098540; A/duck/Australia/341/1983(H15N8), CY006009 (datasets H15-1, H15-2, H15-3);

A/wedge-tailed shearwater/Western Australia/2576/1979(H15N9), CY006010 (dataset H15-1);

A/Australian shelduck/Western Australia/1756/1983(H15N2), CY006032 (dataset H15-2);

A/sooty tern/Western Australia/2327/1983(H15N9), CY006034 (dataset H15-3).

Subtype H16: A/black-headed gull/Sweden/2/99(H16N3), AY684888; A/black-legged kittywake/Alaska/295/1975(H16N3), CY015160; A/glaucous-winged gull/SC Alaska/9JR0783R0/2009 (H16N3), CY070890; A/Fulica atra/Volga/635/1986(H16N3), EU564109; A/common gull/Norway/10_1617/2006(H16N3), FM179755; A/herring gull/Newfoundland/GR032/2010(H16N3), KC845043 (dataset H16-1);

A/mallard/Gurjev/785/83(H16N3), EU148600; A/herring gull/Delaware Bay/712/1988(H16N3), CY005933; A/glaucous gull/Alaska/44198-027/2006(H16N3), HM059998; A/black-headed gull/Turkmenistan/13/76(H16N3), EU293864; A/herring gull/Norway/10_1623/2006(H16N3), FM179756; A/environment/California/1242V/2012(H16N3), CY176997 (dataset H16-2);

A/shorebird/New Jersey/840/1986(H16N3), CY014599; A/gull/SE Alaska/10JR01527R0/2010(H16N3), CY130485; A/teal/Volga/671/86(H16N3), EU148602; A/black-headed gull/Sweden/5/99(H16N3), AY684891; A/shorebird/Delaware/168/06(H16N3), EU030976; A/duck/Hokkaido/WZ82/2013(H16N3), AB937721 (dataset H16-3).

The alignments of representative sequences in each of the datasets were constructed using the Clustal Omega program provided by the EMBL-EBI bioinformatics tool framework[21]. The database entries with missing end sequences, known to be conserved in all influenza strains, were extended using these consensus sequences. This was necessary to avoid potential inaccuracies in structure predictions determined by incomplete input alignments. The same alignments were used for both RNAalifold and RNAz predictions. Both programs were used with default parameters. The RNAalifold algorithm calculates the

secondary structure optimal for a dataset of aligned RNA sequences, based on the ensemble of possible structures. The RNAz calculates a consensus structure in the scanning window (default 120 nucleotides), using a scoring based on structure stability and conservation. The default threshold of this score (P>0.5), which estimates a probability of structure in a window, was used. RNAalifold and RNAz predictions were carried out for both positive- and negative-sense HA RNAs (RNAz includes this as an automatic option).

Three different datasets used for every subtype allowed us to obtain alternative predictions which overlapped only partially. The overlaps were used to identify potential conserved local structures. Such local motifs were selected according to their occurrence in at least two out of three models yielded by any of the two used folding algorithms and putative covariation pattern in the representative sequences from the dataset triplet. Pairs of nucleotides at a distance of more than 50 nucleotides from each other were not considered due to low reliability of predictions of long-range interactions. A putative covariation was considered only if the number of sequences in the three datasets with a double change as compared to the dominant pair was not smaller than the number of sequences with at least one of the corresponding single substitutions. For instance, a case with 12 GC pairs, 2 AC, 2 GU and 2 AU out of 18 combinations was selected, but 10 GC, 3 AC, 3 GU and 2 AU were not considered as a promising covariation candidate. With this relatively smooth threshold, significant correlations were unlikely to be missed, but a number of selected covariations were estimated as non-significant ones at the next step, by mutual information calculations on the basis of all sequences available in GenBank.

Possible RNA structures in the regions containing local motifs conserved in different subtypes were further explored using the Mfold algorithm based on free energy minimization[22]. Both optimal and suboptimal structures were predicted. The Mfold program was also used for free energy calculations.

Mutual information calculations. Mutual information *M(xy)* and the ratios of *M(xy)* and entropies of alignment positions were calculated as described earlier[23,24]. For two putatively paired positions *x* and *y* in the sequence alignment the *M(xy)* value can be calculated as follows:

$$M(xy) = \sum_{b_x,b_y \in (A,G,C,U)} f(b_x b_y) \cdot log_4 \frac{f(b_x b_y)}{f(b_x) f(b_y)} = H(x) + H(y) - H(xy).$$

Here $f(b_x)$, $f(b_y)$ and $f(b_xb_y)$ are frequencies of nucleotides and pairs (b_x, $b_y \in [A,G,C,U]$), the summation is taken over all observed b_xb_y combinations. The entropy values $H = - \Sigma f(b) \cdot log_4 [f(b)]$ describe variations at each of the positions separately and in the pair. The ratios $R_1(xy) = M(xy) / H(x)$ and $R_2(xy) = M(xy) / H(y)$ are more informative than $M(xy)$ in the evaluations of secondary structure models, because they take into account the biased variations at any of the two positions[23,24].

The calculations were performed using the HA sequences retrieved from GenBank using the Influenza Virus Resource[20], accessed in March-July, 2015. Only sequences annotated as "full length only", i.e. containing complete coding regions, were selected. This selection contained 6080 HA sequences of subtype H1, 529 (H2), 10005 (H3), 1412 (H4), 3841 (H5), 1394 (H6), 1984 (H7), 124 (H8), 2028 (H9), 873 (H10), 537 (H11), 162 (H12), 109 (H13), 16 (H14), 13 (H15), and 48 (H16), in total 29155 HA sequences. In order to minimize the influence of the database bias on the computation, the massively sequenced 2009 H1N1 strains with HA segments of classic swine lineage[7] were excluded from $M(xy)$ calculations. The selected sequences were downloaded in separate groups defined by subtype, host, time of isolation and geographical location, allowing us to trace covariation transitions. The sequences in the downloaded fasta format files with less than 1000 sequences (or smaller than 1 Mb) were aligned using the Clustal Omega program for multiple alignment. For larger files the Kalign program was used. Both multiple alignment programs were accessed via the EMBL-EBI bioinformatics tool framework[21].

Confidence levels of correlation values. In order to estimate statististical significance of calculated correlation values, the performance of our protocol of RNA structure predictions was tested on permuted H2 HA sequences. The permutations were done using R language of the Rstudio package[25]. In each of these permutations, all 18 representative sequences of three H2 datasets were reshuffled using the same random vector of numbers between 1 and 1773 (H2 HA segment length), thus yielding three datasets consisting of sequences with the same length, diversity and phylogenetic clustering as real HA segments. The covariations suggested by RNAalifold algorithm predictions in these reshuffled sequences corresponded to pairs of positions in natural H2 HA segments which were not involved in any structure, sometimes located far from each other along the sequence (Supplementary Table S11). Correlation values calculated for pairs yielded by repeated permutation rounds, using all available H2 full-length HA sequences (529 database entries), allowed us to estimate p-values for the correlations in putatively paired positions. The p-values were

defined as probabilities to identify a base pair with a correlation not lower than a specified value in a conserved structural element derived from three datasets of homologous sequences by the method identical to the one used for HA sequences. Repeating the whole procedure 20 times yielded 19 pairs with correlation values of at least 0.5, with two best scores of 0.94 and 0.76 (Table S11). This was sufficient to estimate that the correlations with ratios $R_1(xy)$ and $R_2(xy)$ smaller than 0.8 corresponded to p-values higher than 0.05, thus being non-significant according to the standard significance criterion, and scores of about 0.5 corresponded to p-values (or E-values) close to 1. Apparently, for a putative base pair with correlation values lower than 0.5 it is only reasonable to introduce an E-value (number of base pairs with a correlation not lower than a specified level, expected to be predicted by chance in three datasets of homologous sequences), in this case $E > 1$.

The correlation value of 0.8 seems to be rather strict compared to the correlations observed in the tRNA structure "golden standard" model: 8 out of 21 tRNA base pairs have lower values[23]. Examination of results on permuted HA sequences identified the origin of multiple high correlation values in pairs of positions not constrained by RNA structure: the majority of them were obtained at sites that were characterized by low mutation frequency but yet changed upon HA speciation between Eurasian and American groups of avian strains or between avian and human viruses. This result resembles the one obtained on protein data sets and simulated evolutionary scenarios[26]: a covariation signal may arise from slow evolution of two functionally unrelated sites. In case of HA segments, a large number of nucleotide positions with switched biases between just two-three main clades of a given HA subtype yields a high probability of random incorporation of base pairs with high scores into wrong RNA structure predictions. Thus, as many base pairs in functional HA RNA structures are unlikely to have better correlations than e.g. those in the well-conserved tRNA fold[23], the conserved structures in HA segments cannot be reliably identified by correlation values of single covariations.

Reliability of double covariations in a given structural motif was estimated as the product of their individual occurrence probabilities in the full-length RNA structure predictions multiplied by the probability of two nucleotide pairs to be located in a single structure. Single probabilities were determined as functions of correlation values in the predictions using permuted HA sequences. The probability of two pairs of nucleotides to be located in a structural element can be estimated as approximately the number of all

possible pairs in this structure divided by the number of all possible pairs in the sequence. Bearing in mind that the distance between paired nucleotides is restricted here by 50 nucleotides and the minimal hairpin loop size is 3 nucleotides, the number N_{bp} of all possible pairs in an alignment of length L is easy to calculate as

$$N_{bp} = 47 \times (L - 50) + (47 + 46 + 45 + ... + 1).$$

For HA lengths varying in the range of 1728-1778 nt in different subtypes the N_{bp} value is in the range 8.0×10^4 - 8.2×10^4. Thus, the probability of two covariations with individual correlations of 0.5, corresponding to high individual p-values close to 1, to occur in a single predicted structure with about 50 pairs, can be estimated as about 6×10^{-4}. The occurrence of two covariations with scores as small as 0.3, corresponding to estimated E-values of about 1.5 in our predictions of local conserved structures, is characterized by p-value of about 10^{-3}. Thus double covariations with even rather low correlation values can be considered as a support for predicted hairpins. The same evaluation can be applied to covariations occurring more than once at the same base pair. In this case individual probabilities should be calculated separately in the groups of sequences characterized by independent covariations.

These estimates show that two covariation events in a single local structural element, even characterized by relatively low correlation values, can be considered as a reasonable support for the existence of the structure. Actually these calculations provide a numerical illustration for the empirical rule frequently used in comparative RNA structure modeling based on smaller datasets: two covariations are considerably more reliable evidence for a helix than a single one[27].

Plaque assay statistics. Statistical significance of the plaque assay data was performed using the nonparametric Kruskal-Wallis test with a Dunns post-test, when more than 2 groups were compared. A p-value <0.05 was considered to be significant. Statistical significance was compared to the median plaque size of the WT virus. If 2 groups were compared, the nonparametric Mann-Whitney t-test was performed where p<0.05 was considered significant. All statistical analyses were performed using GraphPad Prism 4.02 for Windows (GraphPad Software, San Diego, CA, U.S.A.). Plaque number was not considered as a measure of virus replication in the used experimental settings.

References

1. Bernhart, S.H., Hofacker, I.L., Will, S., Gruber, A.R. & Stadler, P.F. RNAalifold: improved consensus structure prediction for RNA alignments. *BMC Bioinformatics* **9**, 474 (2008).
2. Gruber, A.R., Findeiß, S., Washietl, S., Hofacker, I.L. & Stadler, P.F. RNAz 2.0:improved noncoding RNA detection. *Pac. Symp. Biocomput.* **2010**, 69-79 (2010).
3. Dugan, V.G. *et al.* The evolutionary genetics and emergence of avian influenza viruses in wild birds. *PLoS Pathog.* **4**, e1000076 (2008).
4. Liu, S. *et al.* Panorama phylogenetic diversity and distribution of type A influenza virus. *PLoS One* **4**, e5022 (2009).
5. Bedford, T. *et al.* Integrating influenza antigenic dynamics with molecular evolution. *Elife* **3**, e01914 (2014).
6. Wu, H.B. *et al.* Genetic characterization of subtype H1 avian influenza viruses isolated from live poultry markets in Zhejiang Province, China, in 2011. *Virus Genes* **44**, 441-449 (2012).
7. Garten, R.J. *et al.* Antigenic and genetic characteristics of swine-origin 2009 A(H1N1) influenza viruses circulating in humans. *Science* **325**, 197-201 (2009).
8. Lindstrom, S.E., Cox, N.J. & Klimov, A. Genetic analysis of human H2N2 and early H3N2 influenza viruses, 1957-1972: evidence for genetic divergence and multiple reassortment events. *Virology* **328**, 101-119 (2004).
9. Smith, D.J. *et al.* Mapping the antigenic and genetic evolution of influenza virus. *Science* **305**, 371-376 (2004).
10. Duan, L. *et al.* Characterization of low-pathogenic H5 subtype influenza viruses from Eurasia: implications for the origin of highly pathogenic H5N1 viruses. *J. Virol.* **81**, 7529-7539 (2007).
11. zu Dohna, H., Li, J., Cardona, C.J., Miller, J. & Carpenter, T.E. Invasions by Eurasian avian influenza virus H6 genes and replacement of the virus' North American clade. *Emerg. Infect. Dis.* **15**, 1040-1045 (2009).
12. Wang, G. *et al.* H6 influenza viruses pose a potential threat to human health. *J. Virol.* **88**, 3953-3964 (2014).
13. Lebarbenchon, C. & Stalknecht, D.E. Host shifts and molecular evolution of H7 avian influenza virus hemagglutinin. *Virology J.* **8**, 328 (2011).
14. Xu, K. *et al.* Isolation and characteriation of an H9N2 influenza virus isolated in Argentina. *Virus Res.* **168**, 41-47 (2012).
15. Kim, H.R. *et al.* Characterization of H10 subtype avian influenza viruses isolated from wild birds in South Korea. *Vet. Microbiol.* 161, 222-228 (2012).
16. Vijaykrishna, D. *et al.* The recent establishment of North American H10 lineage influenza viruses in Australian wild waterfowl and the evolution of Australian avian influenza viruses. *J. Virol.* **87**, 10182-10189 (2013).
17. Karamendin, K. *et al.* Phylogenetic analysis of avian influenza viruses of H11 subtype isolated in Kazakhstan. *Virus Genes* **43**, 46-54 (2011).
18. Wille, M. *et al.* Extensive geographic mosaicism in avian influenza viruses from gulls in the northern hemisphere. *PLoS One* **6**, e20664 (2011).
19. Ramey, A.M. *et al.* Genomic characterization of H14 subtype Influenza A viruses in new world waterfowl and experimental infectivity in mallards (*Anas platyrhynchos*). *PLoS One* **9**, e95620 (2014).
20. Bao, Y. *et al.* The influenza virus resource at the National Center for Biotechnology Information. *J. Virol.* **82**, 596-601 (2008).
21. Li, W. *et al.* The EMBL-EBI bioinformatics and programmatic tools framework. *Nucleic Acids Res.* **43**, W580-W584 (2015).
22. Zuker, M. Mfold web server for nucleic acid folding and hybridization prediction. *Nucleic Acids Res.* **31**, 3406-3415 (2003).

23. Gutell, R.R., Power, A., Hertz, G.Z., Putz, E.J. & Stormo, G.D. Identifying constraints on the higher-order structure of RNA: continued development and application of comparative sequence analysis methods. *Nucleic Acids Res.* **20**, 5785-5795 (1992).
24. Gultyaev, A.P. *et al.* RNA structural constraints in the evolution of the influenza A virus genome NP segment. *RNA Biol.* **11**, 942-952 (2014).
25. RStudio. Available at: http://rstudio.com (Accessed: 6th February 2015).
26. Talavera, D., Lovell, S.C. & Whelan, S. Covariation is a poor measure of molecular coevolution. *Mol. Biol. Evol.* **32**, 2456-2468 (2015).
27. Woese, C.R., Gutell, R., Gupta, R. & Noller,H.F. Detailed analysis of the higher-order structure of 16S-like ribosomal ribonucleic acids. *Microbiol. Rev.* **47**, 621-669 (1983).
28. Perdue, M.L., Garcia, M., Senne, D & Fraire, M. Virulence-associated sequence duplication at the hemagglutinin cleavage site of avian influenza viruses. *Virus Res.* **49**, 173-186 (1997).
29. Smith, G.J.D. *et al.* Nomenclature updates resulting from the evolution of avian influenza A(H5) virus clades 2.1.3.2a, 2.2.1, and 2.3.4 during 2013-2014. *Influenza Other Respir. Viruses* **9**, 271-276 (2015).
30. Okamatsu, M. *et al.* Low pathogenicity H5N2 avian influenza outbreak in Japan during the 2005-2006. *Vet. Microbiol.* 124, 35-46 (2007).
31. Lee, C.C.D. et al. Emergence and evolution of avian H5N2 influenza viruses in chickens in Taiwan. *J. Virol.* **88**, 5677-5686 (2014).

SUPPLEMENTARY FIGURES

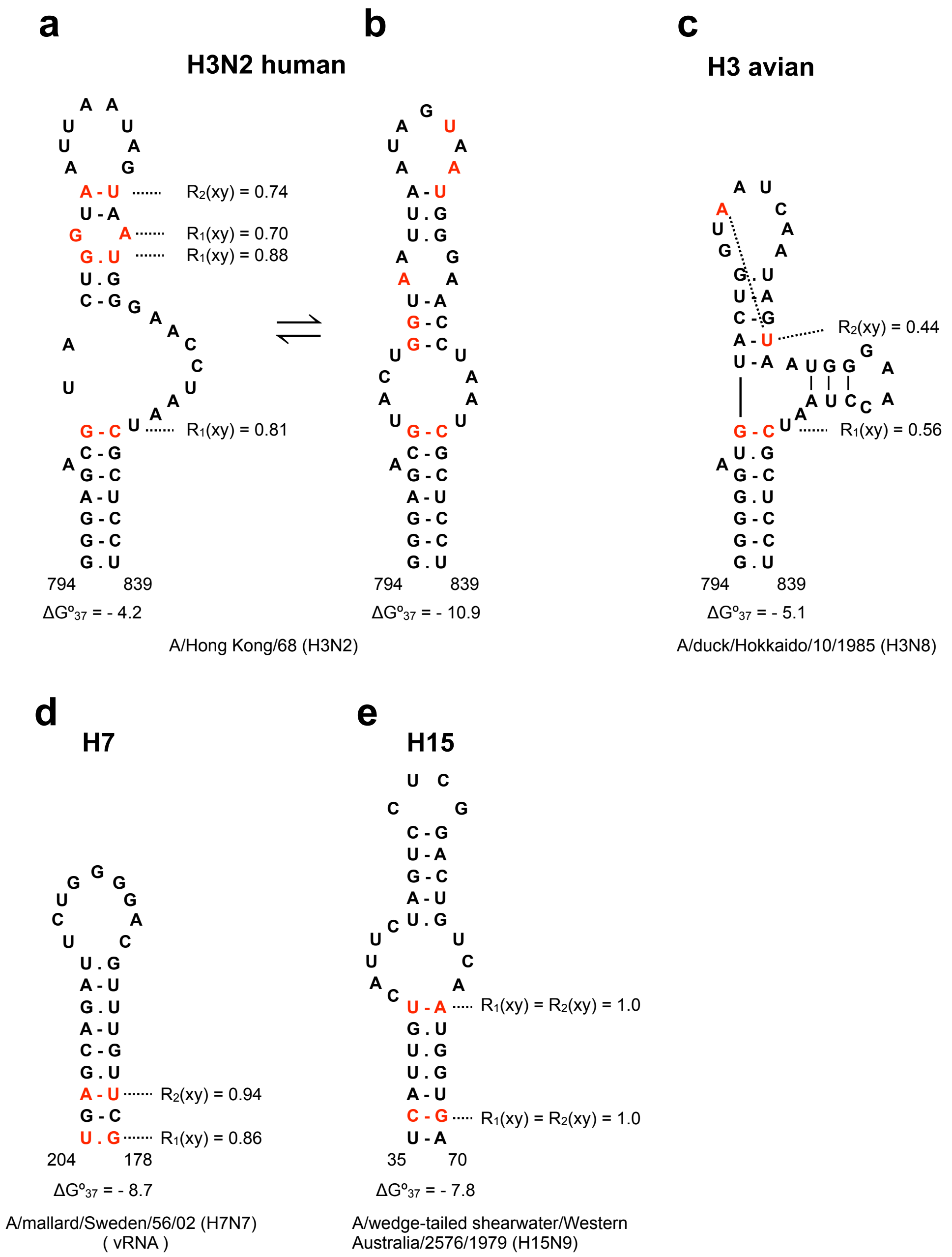

Supplementary Figure S1. Predicted structures containing more than one covariation and their covariation scores. The structures are shown for representative strains. The structured domain 794-839 in HA segments from human H3N2 viruses is supported by four covariations (**a**), although in some strains an alternative conformation (**b**) is thermodynamically more stable. Another conformation of this domain was yielded by the RNAalifold consensus structure predictions in avian H3 HA segments; two out of four human H3N2 covariations are noted in avian H3 viruses (**c**). The H7 HA structure (**d**) was predicted only in the negative-sense vRNA, the corresponding positive-sense structure is less likely because of many CA mismatches. The H15 HA structure (**e**) contains two covariations with perfect correlations which are probably determined by small number of available sequences (N=13). Folding free energies ΔG^{o}_{37} (kcal/mol) are given for positive-sense RNA (**a-c, e**) and for negative-sense vRNA (**d**) .

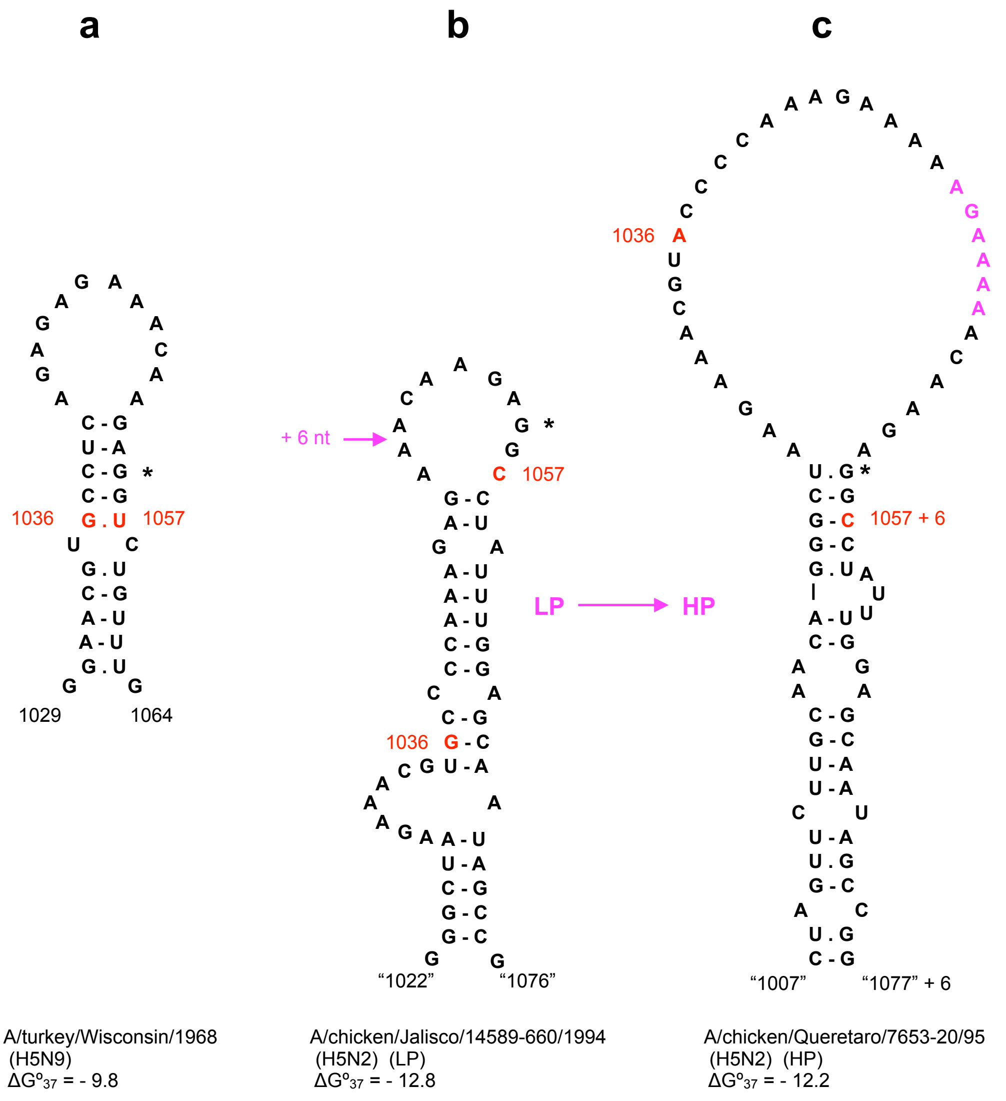

Supplementary Figure S2. Alternative structures in the cleavage site region of H5 HA segments of American origin. The GGN codon coding for the first Gly residue of the HA2 chain downstream of the cleavage site is shown by an asterisk. (**a**) The hairpin structure homologous to those predicted in Eurasian H5 HA segments (Fig. 3b), with nucleotides of the 1036-1057 covariation shown in red. In contrast to Eurasian HA segments, extension of this hairpin is not thermodynamically stable. (**b,c**) Different alternative structures in the lineage of Mexican H5N2 viruses evolving towards highly pathogenic (HP) strains. Domain 1022-1076 (**b**) is locally the lowest free energy conformation in a low pathogenic (LP) strain, also present in the global lowest free energy prediction for this strain[28]. In the HP HA segment containing two additional basic amino acid codons, the most favorable locally stable conformation is different (**c**). The insertion in the LP hairpin and insertion sequence in the HP hairpin are shown in magenta. Numbering of nucleotides corresponds to the full-length LP H5 HA segments. Folding free energies ΔG°_{37} (kcal/mol) are given for positive-sense RNA.

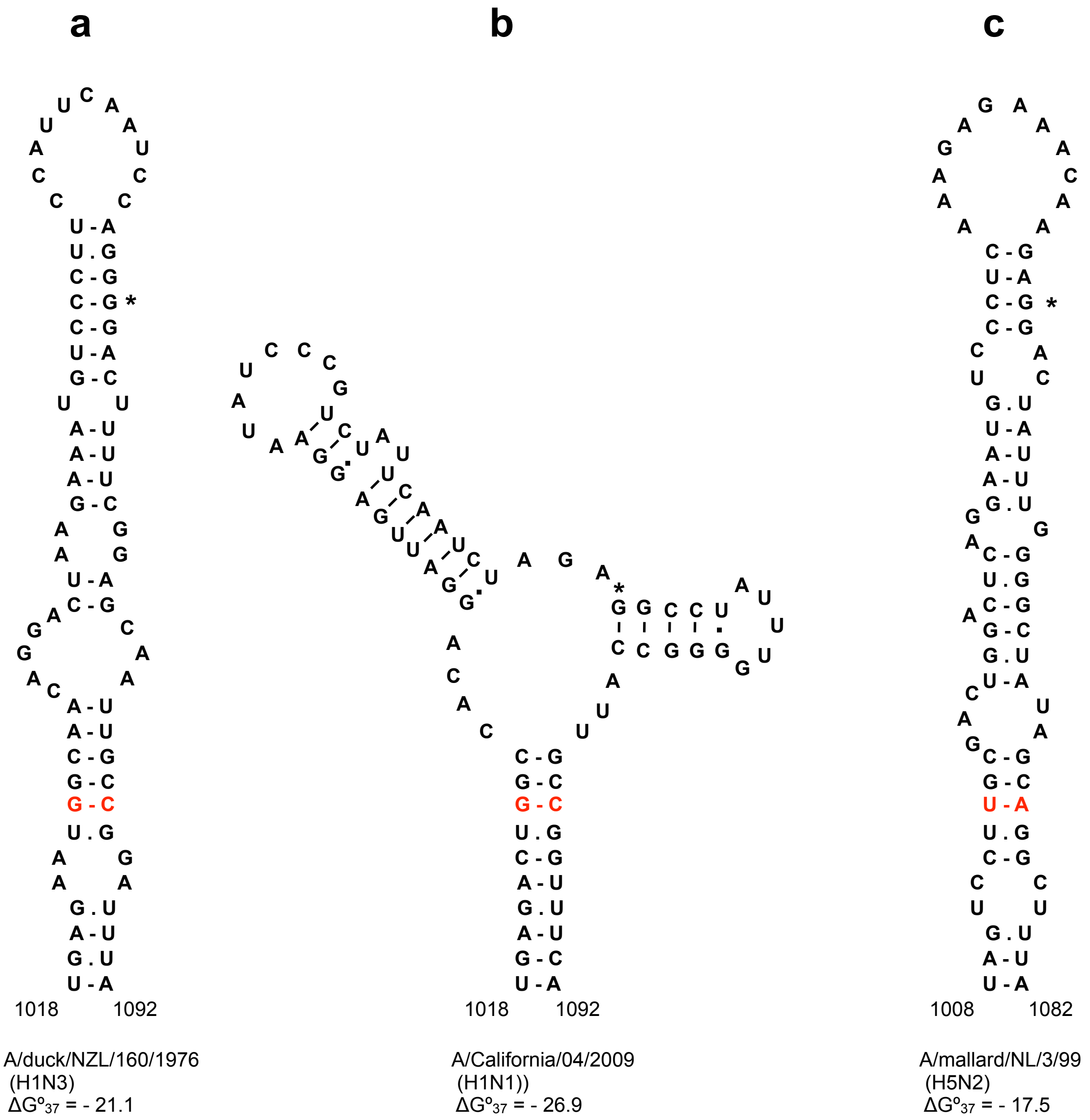

Supplementary Figure S3. Examples of alternative conformations in the domain folded in the cleavage site region of H1 HA segments. The closing stem of the domain is conserved in both topologies (**a**,**b**) and contains the base pair displaying a covariation (shown in red) in the H5 HA stem-loop (**c**). The GGN codon coding for the first Gly residue of the HA2 chain downstream of the cleavage site is shown by an asterisk. Folding free energies ΔG°_{37} (kcal/mol) are given for positive-sense RNA.

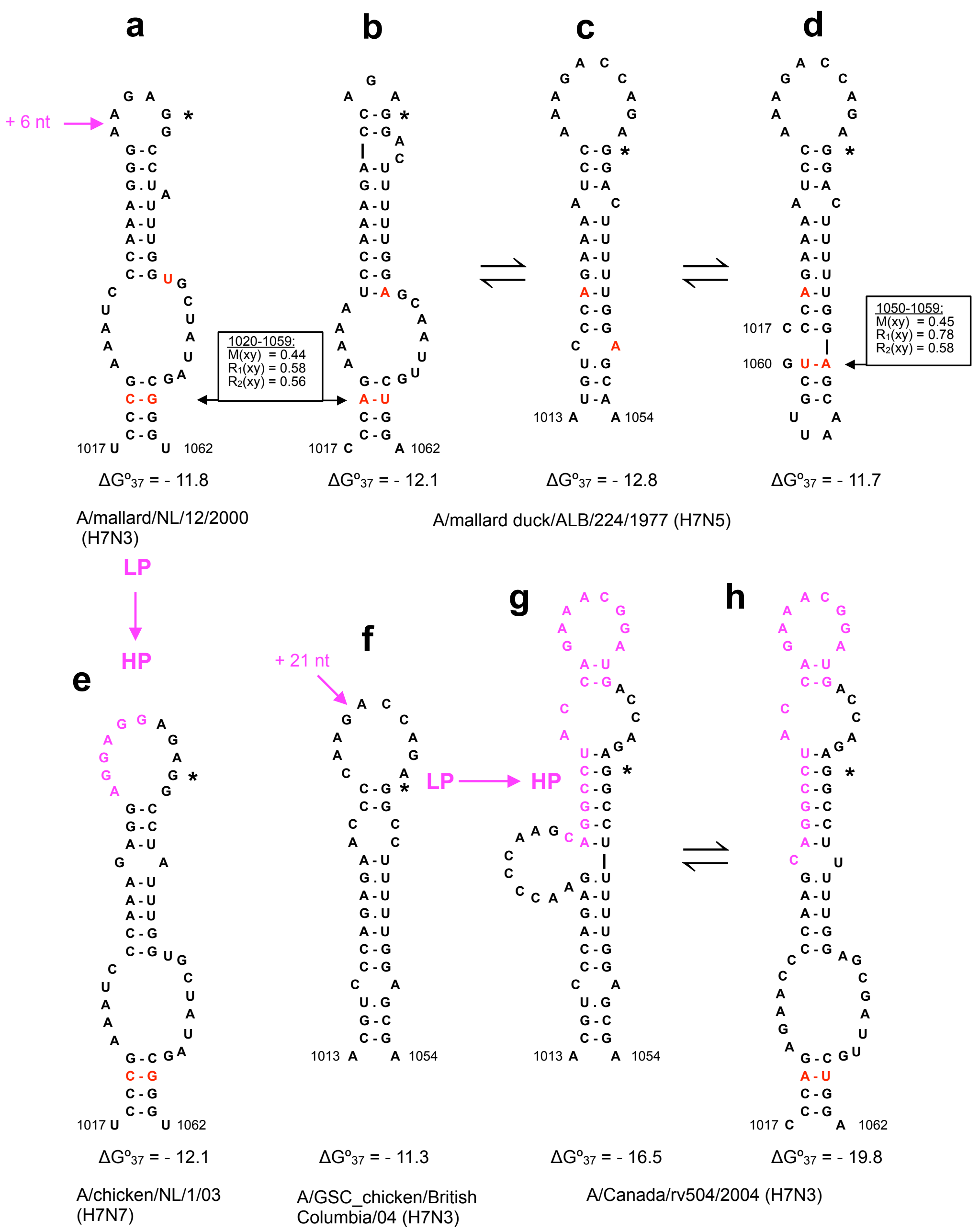

Supplementary Figure S4. Alternative structures in the cleavage site regions of H7 HA segments. (**a,b**) The conformation conserved in both Eurasian and American lineages is supported by a weak covariation 1020-1059 in the closing stem, which is incompatible with small hairpin 1050-1059, supported by more significant covariation (see Fig. 2; Table S1). (**b,c,d**) In many strains alternative structures have very close values of free energy, each of the shown three topologies has the lowest free energy in some strains. (**e,f,g,h**) Insertions leading to the creation of a multibasic cleavage site in HP viruses occur in the loops of the hairpins flanking the insertion sites; the insertions result in similar stem-loop structures which may be refolded into one of the alternatives. The GGN codon coding for the first Gly residue of the HA2 chain downstream of the cleavage site is shown by an asterisk. The covarying nucleotides 1020, 1050 and 1059 are shown in red. Folding free energies ΔG^{o}_{37} (kcal/mol) are given for positive-sense RNA.

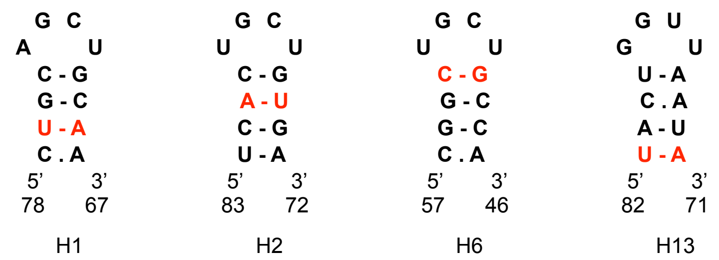

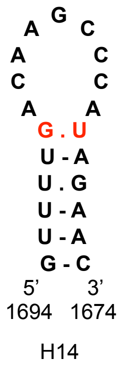

subtype	x,y	M(xy)	R_1(xy)	R_2(xy)
H1	68,77	0.21	0.62	0.59
H2	74,81	0.01	0.07	0.31
H6	49,54	0.15	0.30	0.30
H13	71,82	0.38	0.85	0.85
H14	1679,1689	0.45	1.0	1.0

Supplementary Figure S5. The hairpins predicted in the minimal packaging signal regions of HA vRNA and the scores of covariations in them.

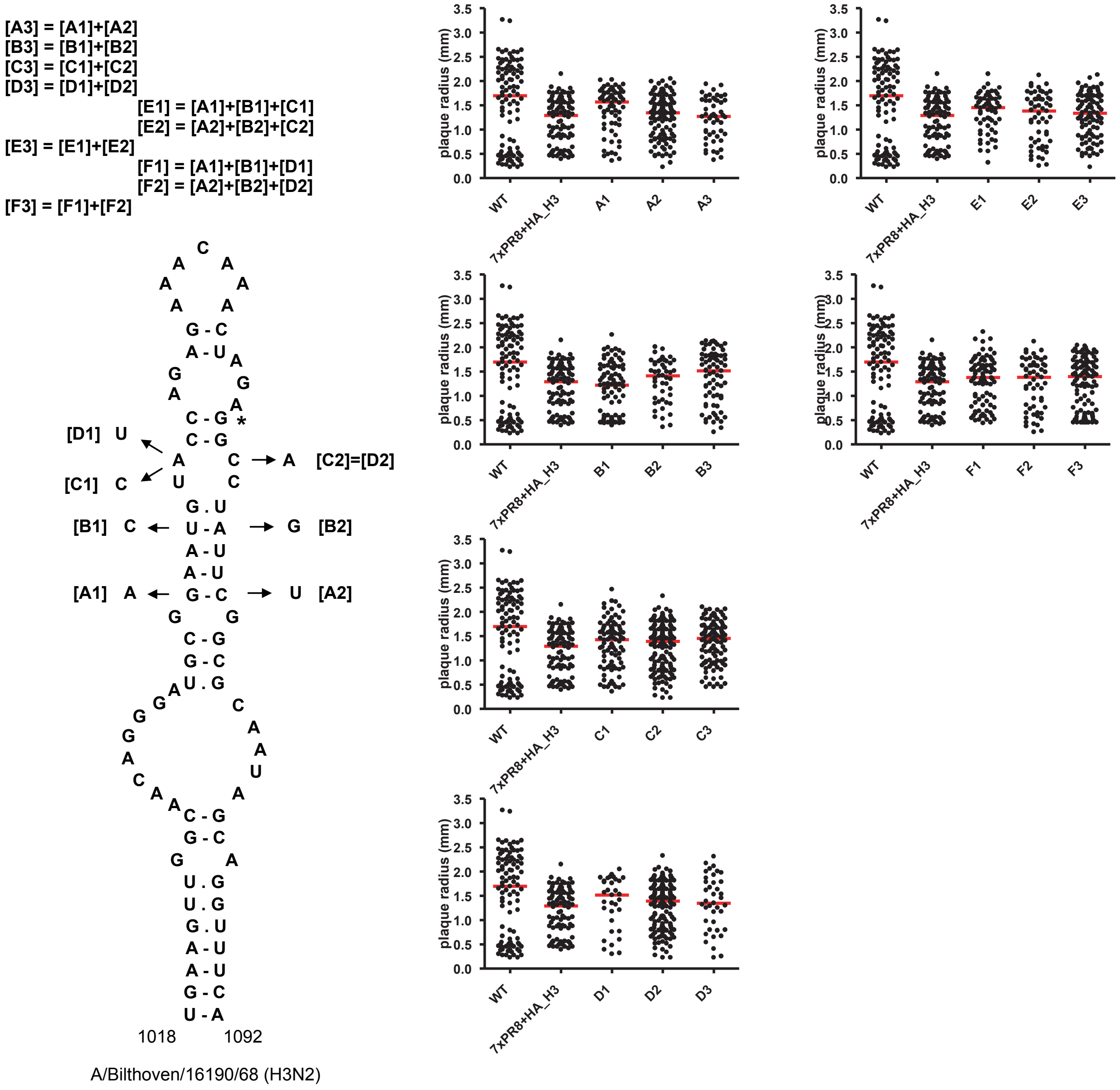

Supplementary Figure S6. Mutagenesis of the predicted HA segment domain 1018-1092 of human H3N2 viruses. The mutagenesis strategy and notations are similar to those described in Fig. 6. Mutant series A, B, C, and D affected one of the base pairs in the domain, mutant series E and F affected 3 pairs. All substitutions are silent. Mutations [C1], [C2] and [C3] reconstruct the covariation observed at the homologous base pair in the H5 HA segments (Fig. 3a,b). WT, A/PR/8/34 virus; 7xPR8+HA_H3, recombinant virus with the A/Bilthoven/16190/68 (H3N2) HA segment. The GGN codon coding for the first Gly residue of the HA2 chain downstream of the cleavage site is indicated by asterisk.

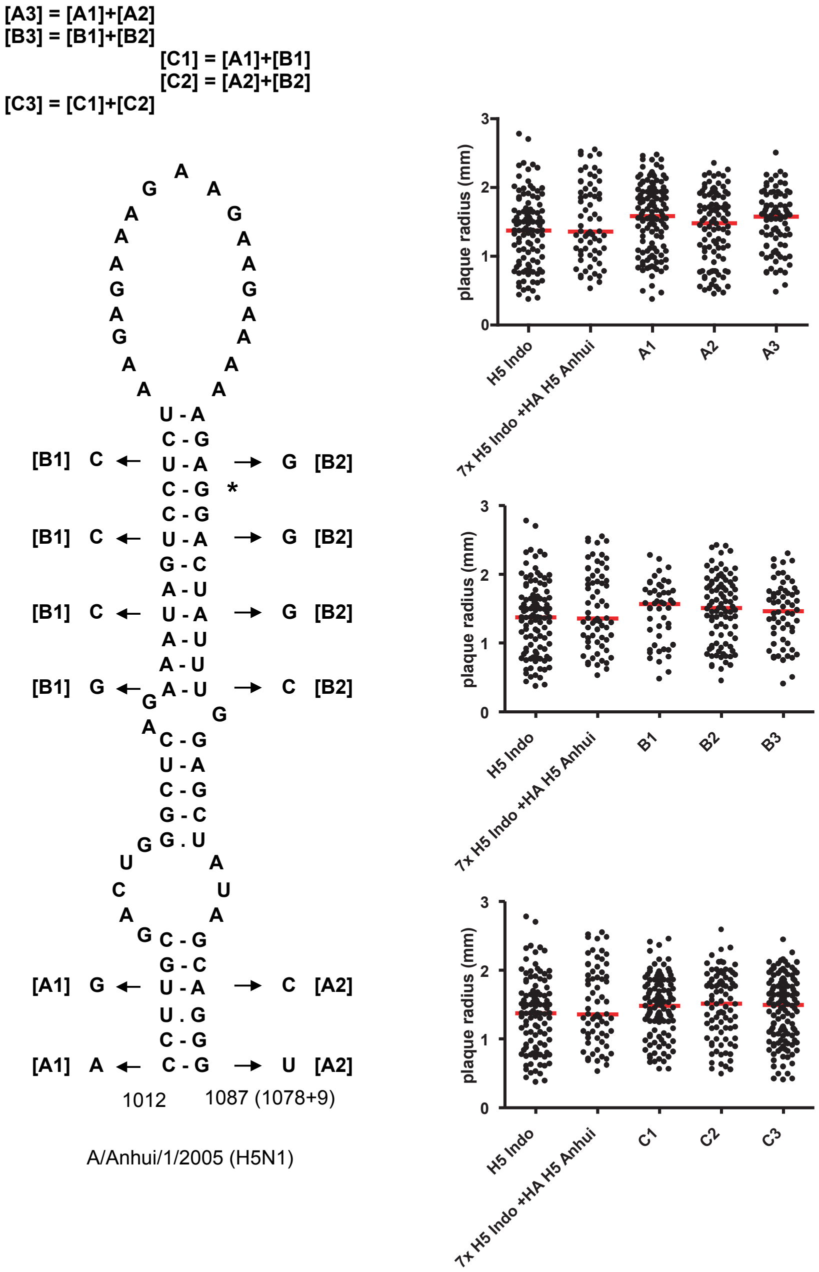

Supplementary Figure S7. Mutagenesis of the predicted HA segment domain 1012-1087 of highly pathogenic H5N1 viruses. The mutagenesis strategy and notations are similar to those described in Fig. 6. Mutant series A affected two of the base pairs in the domain, mutant series B affected 4 pairs, and series C affected 6 pairs. All substitutions are silent. The HA segment of strain A/Anhui/1/2005 (H5N1), used in these experiments, contains an insertion of 9 nucleotides in the loop of the structure. H5 Indo, A/Indonesia/5/05 (H5N1) virus; 7xH5 Indo +HA H5 Anhui, recombinant virus with the A/Anhui/1/2005 (H5N1) HA segment. The GGN codon coding for the first Gly residue of the HA2 chain downstream of the cleavage site is indicated by asterisk.

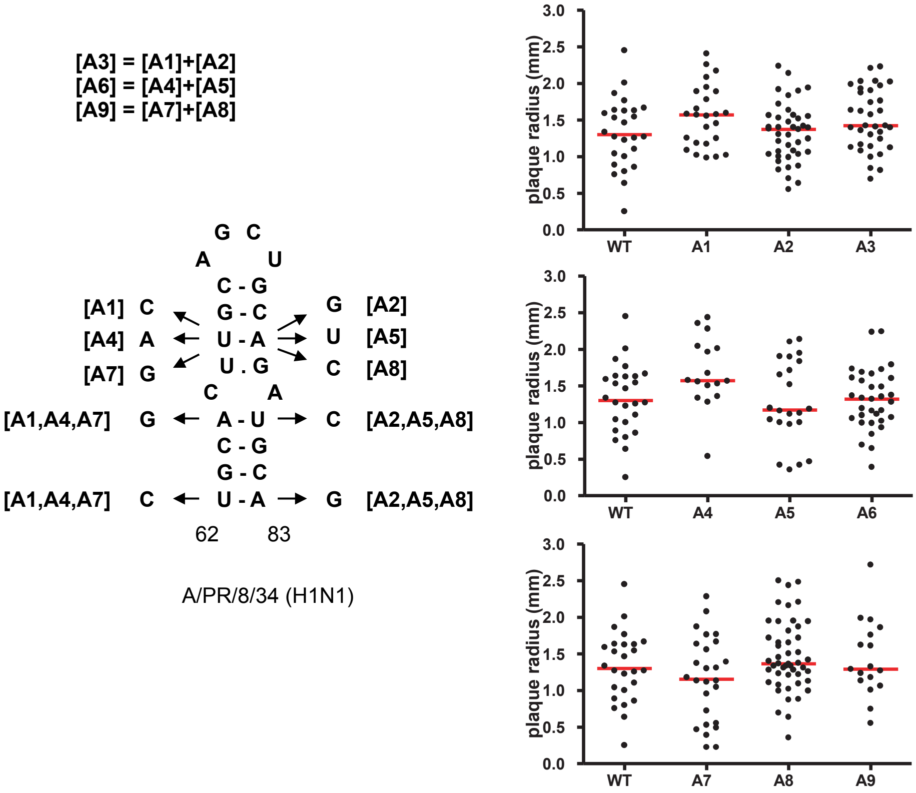

Supplementary Figure S8. Mutagenesis of the predicted hairpin 62-83 in the packaging signal region of H1 HA segments. The mutagenesis strategy and notations are similar to those described in Fig. 6. Each of the mutant series A1-A2-A3; A4-A5-A6 and A7-A8-A9 affected three of the base pairs in the hairpin. In addition to mutations in the hairpin 67-78 (Supplementary Fig. S5), mutations were also introduced in its potential extension 62-83. Such an extension is conserved in a number of H1 and H2 HA segments. All substitutions are silent. Mutations were introduced into the HA gene segment of A/PR/8/34 (H1N1).

SUPPLEMENTARY TABLES

Supplementary Table S1. Base pairs in the predicted local structures with at least one correlation value of 0.5 or more. The perfect correlations $R_1(xy)=R_2(xy)=1.0$ in the H14 and H15 subtypes are determined by small numbers of available HA sequences (16 and 13 strains, respectively) that do not contain mismatches at the paired positions. Host specificity is indicated if a structure is predicted only in one of host-specific datasets of a given subtype.

subtype	positions	M(xy)	R_1(xy)	R_2(xy)	(host)
H1	68-77	0.21	0.62	0.59	
	727-776	0.05	0.57	0.43	human
	1592-1603	0.33	0.65	0.66	swine
H2	925-934	0.31	0.63	0.63	
	1438-1450	0.23	0.56	0.65	
H3	801-833	0.03	0.81	0.62	human
	806-821	0.09	0.88	0.60	human
	807-820	0.05	0.70	0.65	human
	809-818	0.06	0.65	0.74	human
	1040-1073	0.31	0.90	0.90	human
H4	1043-1060	0.40	0.75	0.66	
	1045-1054	0.41	0.86	0.48	
	1336-1348	0.36	0.74	0.49	
H5	133-148	0.20	0.56	0.28	
	1036-1057	0.26	0.36	0.59	
	1426-1432	0.22	0.63	0.56	
H6	1070-1094	0.28	0.54	0.79	
H7	153-162	0.41	0.62	0.73	
	178-204	0.41	0.86	0.66	
	180-202	0.46	0.75	0.94	
	378-386	0.29	0.53	0.60	
	1050-1059	0.45	0.78	0.58	
	1143-1167	0.36	0.60	0.48	
	1221-1231	0.41	0.86	0.72	
H8	166-175	0.50	0.65	0.61	
	787-793	0.23	0.55	0.48	
	999-1033	0.38	0.88	0.56	
	1186-1192	0.28	0.67	0.67	
	1549-1555	0.37	0.89	0.87	
	1588-1600	0.37	0.86	0.86	
H10	1051-1060	0.48	0.74	0.63	
	1222-1234	0.49	0.67	0.89	
	1300-1306	0.48	0.87	0.80	
H11	266-275	0.39	0.70	0.63	
	288-299	0.30	0.71	0.69	
	452-461	0.43	0.74	0.73	
	467-479	0.40	0.73	0.84	
	1382-1397	0.35	0.83	0.75	
H13	71-82	0.38	0.85	0.85	
	331-339	0.28	0.62	0.58	
	639-651	0.52	0.68	0.66	
	1587-1593	0.44	0.90	0.65	
	1616-1630	0.24	0.66	0.65	
H14	165-208	0.48	1.0	1.0	
	650-668	0.45	1.0	1.0	
	1166-1181	0.45	1.0	1.0	
	1169-1175	0.45	1.0	1.0	
	1400-1434	0.45	1.0	1.0	
	1679-1689	0.45	1.0	1.0	
H15	36-69	0.31	1.0	1.0	
	41-64	0.31	1.0	1.0	
	842-867	0.31	1.0	1.0	

Supplementary Table S2. Number of HA sequences of human H3N2 strains in GenBank with various combinations of nucleotides 801/833. Covariation scores: M(xy) = 0.03; R_1(xy) = 0.81; R_2(xy) = 0.62.

isolation year	GC	AC	GU	AU	UU	AA
1968-1970	52	-	-	1	-	-
1971	7	-	-	3	-	-
1972	2	-	-	20	-	-
1973-1976	-	-	-	51	-	-
1977-1978	1	-	4	15	-	-
1979-now	-	5	1	8285	2	20
total	62	5	5	8375	2	20

Supplementary Table S3. Number of HA sequences of human H3N2 strains in GenBank with various combinations of nucleotides 806/821. Covariation scores: M(xy) = 0.09; R_1(xy) = 0.88; R_2(xy) = 0.60.

isolation year	GU	GC	UA	CA	UG	UC	UU	GA
1968-1985	202	2	-	-	-	-	1	-
1986-1988	12	17	-	-	-	-	-	-
1989-1990	2	16	-	-	-	15	-	-
1991	-	2	-	-	-	36	-	-
1992	-	-	9	-	-	30	-	-
1993	-	-	100	-	-	5	-	-
1994-now	2	-	7995	3	1	14	3	2
total	218	37	8104	3	1	100	4	20

Supplementary Table S4. Number of HA sequences of human H3N2 strains in GenBank with various combinations of nucleotides 807/820. Covariation scores: M(xy) = 0.05; R_1(xy) = 0.70; R_2(xy) = 0.65.

isolation year	UA	UG	CA	UC	UU	CC	GA	AA
1968-1970	-	-	-	-	-	-	53	-
1971-1973	-	-	-	-	-	-	37	5
1974-1975	4	-	-	-	-	-	19	-
1976-1981	29	2	-	-	-	-	22	-
1982	-	2	1	7	-	-	1	-
1983-1985	-	1	1	20	1	-	-	-
1986-1990	-	-	-	61	1		-	-
1991-now	3	-	-	8167	4	20	1	1
total	36	5	2	8255	6	20	133	6

Supplementary Table S5. Number of HA sequences of human H3N2 strains in GenBank with various combinations of nucleotides 809/818. Covariation scores: M(xy) = 0.06; $R_1(xy)$ = 0.65; $R_2(xy)$ = 0.74.

isolation year	AU	AC	GU	GC
1968-1981	168	3	-	
1982-1983	6	11	-	
1984	-	4	-	
1985-1986	-	9	-	13
1987-1999	-	-	-	795
2000-now	1	23	16	7420
total	175	50	16	8228

Supplementary Table S6. Number of HA sequences of H7, H10 and H15 strains in GenBank with various combinations of nucleotides corresponding to 1050/1059 in H7 subtype.
Covariation scores: M(xy) = 0.47; $R_1(xy)$ = 0.76; $R_2(xy)$ = 0.58.

origin	UA	UG	CA	CG	AU	GU	AC	GC	AA	GA	UU	CU
H7 Eurasia	293	918	5	11	1	-	-	-	-	-	3	-
America	-	3	-	1	631	39	30	-	2	-	-	-
Oceania	1	-	-	-	-	-	-	-	-	-	-	16
equine	23	-	-	-	-	-	-	-	-	-	-	-
H10 Eurasia	244	132	-	-	1	-	-	-	-	-	-	-
America	-	1	-	-	421	51	12	1	1	6	-	-
Oceania	1	-	-	-	-	-	-	-	-	-	-	-
H15	11	-	2	-	-	-	-	-	-	-	-	-
total	573	1054	7	12	1054	90	42	1	3	6	3	16

Supplementary Table S7. Number of HA sequences of human H3N2 strains in GenBank with various combinations of nucleotides 1040/1073. Covariation scores: M(xy) = 0.31; $R_1(xy)$ = 0.90; $R_2(xy)$ = 0.90.

isolation year	GC	AC	GU	AU	UC	AG
1968-2001	1263	13	1	-	1	-
2002	221	-	-	17	-	-
2003	16	2	-	471	-	-
2004-now	29	24	34	6377	-	1
total	1529	39	35	6865	1	1

Supplementary Table S8. Number of HA sequences of H5 strains in GenBank with various combinations of nucleotides 1036/1057. Covariation scores: M(xy) = 0.26; R_1(xy) = 0.36; R_2(xy) = 0.59.

origin	UA	CA	UG	CG	GC	GU	AC	AU	UC	CC	CU	UU	AA
Asia	952	1206	45	13	-	1	33[c]	3[c]	-	-	62	8	3
Europe	16	299	5	11	-	-	-	-	-	-	8	5	-
Africa	26	611	1	2	-	-	-	-	-	-	6	-	-
Oceania	-	1	-	-	-	-	-	-	1	1	-	1	-
America then-2012	-	1[d]	-	-	25	49	373	12	-	2	-	-	11
2013	33[a]	-	-	-	-	-	1	-	-	-	-	-	-
2014	11[b]	-	-	-	-	-	-	-	1	-	-	-	-
total	1038	2118	51	26	25	50	407	15	2	3	76	14	14

[a] Separate covariation event to the Eurasian-like UA pair in 33 Californian strains of 2013. The non-random character of this clade-specific covariation is supported by BLAST searches showing that the Californian 2013 HA segments are most closely related to two HA segments with a CC mismatch, which are unique in America.

[b] The UA-containing HA segments isolated from American strains in 2014 are of Asian origin[29] and cannot be considered as an independent covariation.

[c] Both BLAST and literature data[30,31] showed that the AC- and AU-containing HA segments from Asian strains belong to the American lineage and were not a covariation.

[d] American strain A/emu/NY/12716/1994 (H5N9) is of European origin (BLAST).

Supplementary Table S9. Number of HA sequences of H5 strains in GenBank with various combinations of nucleotides 1009/1081. Covariation scores: M(xy) = 0.17; R_1(xy) = 0.54; R_2(xy) = 0.50.

origin	AU	GU	AC	GC	AA	AG
Asia	2121	49	125	31	-	-
Europe	293	8	29	13	-	-
Africa	627	8	7	-	1	2
Oceania	1	2	-	1	-	-
America	11	20	4	485	-	-
total	3053	87	165	530	1	2

Supplementary Table S10. Number of HA sequences of H1, H2, H5 and H6 strains in GenBank with various combinations of nucleotides homologous to 1010/1070 positions in H6 HA segments. Covariation scores: M(xy) = 0.32; R_1(xy) = 0.67; R_2(xy) = 0.61 within H6 strains; M(xy) = 0.45; R_1(xy) = 0.75; R_2(xy) = 0.56 in the whole H5/H6/H2/H1 cluster.

subtype	GC	GU	AC	AU	UA	UG	CA	CG	AA	GA	GG	UU	UC	CU
H1	3993	2053	-	3	-	-	-	-	-	23	2	1	-	-
H2	64	287	-	175	-	-	-	-	-	3	-	-	-	-
H5	-	-	10	-	3599	19	50	3	50	1	-	12	94	1
H6 Asia	-	-	-	-	645	70	1	-	4	-	-	-	1	-
Europe	-	-	-	-	39	2	-	-	-	-	-	-	-	-
Africa	-	2	-	-	4	-	-	-	-	-	-	-	-	-
Oceania	-	4	-	-	7	-	-	-	-	2	-	-	-	-
America	-	82	-	152	324	34	15	-	-	1	-	-	-	1
total	4057	2428	10	330	4618	125	66	3	54	30	2	13	95	2

Supplementary Table S11. Pairs of positions in H2 HA sequences with at least one correlation value of 0.5 or more, yielded by the RNAalifold predictions in the permutation rounds of HA datasets. Position numbering corresponds to that in real H2 HA sequences. The pairs are given in descending order of the highest score for a given pair. P-value estimates were calculated as numbers of covariations with scores higher or equal to a given score, divided by the number of permutation rounds (20).

positions	M(xy)	R_1(xy)	R_2(xy)	max[R_1(xy),R_2(xy)]	p-value estimate
450-421	0.47	0.92	0.94	0.94	$\lesssim$0.05
637-196	0.56	0.76	0.73	0.76	0.1
742-196	0.53	0.75	0.69	0.75	0.2
1162-236	0.38	0.67	0.75	0.75	0.2
148-1633	0.37	0.74	0.74	0.74	0.25
1652-1303	0.35	0.69	0.68	0.69	0.35
736-310	0.39	0.52	0.69	0.69	0.35
145-148	0.34	0.60	0.68	0.68	0.4
78-891	0.33	0.66	0.66	0.66	0.45
1280-1249	0.32	0.65	0.65	0.65	0.5
789-344	0.31	0.63	0.64	0.64	0.55
1652-1354	0.31	0.61	0.62	0.62	0.6
700-798	0.31	0.57	0.61	0.61	0.7
1468-334	0.30	0.61	0.61	0.61	0.7
262-345	0.46	0.59	0.57	0.59	0.75
904-1162	0.29	0.58	0.51	0.58	0.8
1522-928	0.20	0.54	0.39	0.54	0.85
1390-331	0.26	0.52	0.53	0.53	0.9
463-226	0.26	0.52	0.51	0.52	0.95
